# The landscape of drug resistance in *Plasmodium falciparum* malaria in the Democratic Republic of Congo: a mapping systematic review

**DOI:** 10.1186/s41182-023-00551-7

**Published:** 2023-11-15

**Authors:** Nadine Kalenda Kayiba, Evariste Tshibangu-Kabamba, Angel Rosas-Aguirre, Natsuko Kaku, Yu Nakagama, Akira Kaneko, Dieudonné Mvumbi Makaba, Doudou Yobi Malekita, Brecht Devleesschauwer, Joris Losimba Likwela, Pius Kabututu Zakayi, Patrick DeMol, Georges Mvumbi Lelo, Marie-Pierre Hayette, Paul Lusamba Dikassa, Yasutoshi Kido, Niko Speybroeck

**Affiliations:** 1 Research Institute of Health and Society, Université Catholique de Louvain, Brussels, Belgium; 2 Department of Public Health, Faculty of Medicine, University of Mbujimayi, Mbujimayi, Democratic Republic of Congo; 3 Research Center for Infectious Disease Science & Department of Virology and Parasitology, Graduate School of Medicine, Osaka Metropolitan University, Osaka, Japan; 4 Department of Internal Medicine, Faculty of Medicine, University of Mbujimayi, Mbujimayi, Democratic Republic of Congo; 5 Department of Basic Sciences, Faculty of Medicine, University of Kinshasa, Kinshasa, Democratic Republic of Congo; 6 Department of Quality of Laboratories, Sciensano, Brussels, Belgium; 7 Department of Epidemiology and Public Health, Sciensano, Brussels, Belgium; 8 Department of Translational Physiology, Infectiology and Public Health, Ghent University, Merelbeke, Belgium; 9 Department of Public Health, Faculty of Medicine, University of Kisangani, Kisangani, Democratic Republic of Congo; 10 Laboratory of Clinical Microbiology, Center for Interdisciplinary Research on Medicines, University of Liège, Liège, Belgium; 11 School of Public Health, Faculty of Medicine, University of Kinshasa, Kinshasa, Democratic Republic of Congo

**Keywords:** Democratic Republic of Congo, Malaria, Antimalarial drug resistance, Mutations, Molecular markers of drug resistance

## Abstract

**Context:**

The Democratic Republic of Congo (DRC), one of the most malaria-affected countries worldwide, is a potential hub for global drug-resistant malaria. This study aimed at summarizing and mapping surveys of malaria parasites carrying molecular markers of drug-resistance across the country.

**Methods:**

A systematic mapping review was carried out before July 2023 by searching for relevant articles through seven databases (PubMed, Embase, Scopus, African Journal Online, African Index Medicus, Bioline and Web of Science).

**Results:**

We identified 1541 primary studies of which 29 fulfilled inclusion criteria and provided information related to 6385 *Plasmodium falciparum* clinical isolates (collected from 2000 to 2020). We noted the *Pf*CRT K76T mutation encoding for chloroquine-resistance in median 32.1% [interquartile interval, IQR: 45.2] of analyzed malaria parasites. The proportion of parasites carrying this mutation decreased overtime, but wide geographic variations persisted. A single isolate had encoded the *Pf*K13 R561H substitution that is invoked in artemisinin-resistance emergence in the Great Lakes region of Africa. Parasites carrying various mutations linked to resistance to the sulfadoxine–pyrimethamine combination were widespread and reflected a moderate resistance profile (*Pf*DHPS A437G: 99.5% [IQR: 3.9]; *Pf*DHPS K540E: 38.9% [IQR: 47.7]) with median 13.1% [IQR: 10.3] of them being quintuple IRN–GE mutants (i.e., parasites carrying the *Pf*DHFR N51I–C59R–S108N and *Pf*DHPS A437G–K540E mutations). These quintuple mutants tended to prevail in eastern regions of the country. Among circulating parasites, we did not record any parasites harboring mutations related to mefloquine-resistance, but we could suspect those with decreased susceptibility to quinine, amodiaquine, and lumefantrine based on corresponding molecular surrogates.

**Conclusions:**

Drug resistance poses a serious threat to existing malaria therapies and chemoprevention options in the DRC. This review provides a baseline for monitoring public health efforts as well as evidence for decision-making in support of national malaria policies and for implementing regionally tailored control measures across the country.

**Supplementary Information:**

The online version contains supplementary material available at 10.1186/s41182-023-00551-7.

## Background

The Democratic Republic of Congo (DRC) has always been highly endemic for *Plasmodium falciparum* malaria [1, 2]. Until the middle of the twentieth century, quinine—i.e., the first drug used for malaria treatment and prophylaxis, was not supplied by extensive programs due to reduced availability and high cost of its importation from South–East Asia [1–3]. The World War II prompted colonial authorities to start producing the drug locally and to introduce newly developed synthetic antimalarial products, namely, chloroquine and pyrimethamine [1, 2, 4]. Due to its low-cost and high efficacy, chloroquine quickly became a leading antimalarial drug enabling large-scale distribution programs through a few urban and industrial cities that existed in the country in 1940s–1950s [1]. However, the efforts initiated by the colonial administration to fight against malaria were prematurely interrupted following the accession to independence of the country in 1960, which led to the hasty departure of the colonial health officials and the rapid dismantling of the Congolese health system hitherto under construction [3, 5]. Malaria control activities were relaunched in the early 1970s at the scale of Kinshasa (i.e., the country's capital city) before being extended to the entire country in the 1980s [5–8]. Finally, the National Malaria Control Program (NMCP) was only created in 1998 to address malaria with broad mitigation efforts and health policies [6–8].

Therefore, despite a relatively recent introduction of modern antimalarial drugs, malaria control efforts have often been carried out outside any strong policies and regulatory frameworks in the country. The history of malaria control has consequently been dominated by low adoption of official policies as well as popular practices dominated by self-medication, consumption of herbal medicines, and over-the-counter access to drugs of questionable quality [2, 6, 9, 10]. The resulting high drug abuse has potentially served as a setting for the emergence or spread of drug resistance. In this context, the historical effectiveness of chloroquine against malaria could not be sustained for long in the country. Chloroquine-resistant malaria, first suspected in early 1980s [11], evolved rapidly and was already widespread and associated with excessive malaria morbidity and mortality across the country by the time the NMCP was created [12, 13]. Since then, the NMCP has primarily focused on adjusting strategies to the evolving landscape of drug-resistant malaria and scaling up antimalarial activities nationwide [6–8, 13–18]. Chloroquine was thus replaced by sulfadoxine–pyrimethamine (S–P) in 2002 followed by artemisinin-based combination therapies (ACTs) in 2005 [6, 8, 13] as first-line recommended treatment for uncomplicated malaria. Currently used ACTs include artesunate–amodiaquine (since 2005) [8], artemether–lumefantrine (since 2011) [6, 15, 19], and artesunate–pyronaridine (since 2021) [16], alternatively. Unlike chloroquine, which was completely removed from national guidelines, the S–P combination has been limited to intermittent preventive treatment of malaria (IPT) in pregnancy after withdrawal from curative use [6, 15]. Quinine has been dedicated to specific clinical forms of malaria (e.g., severe malaria, malaria in early pregnancy, malaria rescue therapy, malaria in young children) in an attempt to restrict its use and possibly prevent it from drug resistance emergence [6, 13, 16]. Further shifting of quinine away from the frontline treatments has been recently achieved with the introduction of injectable artesunate as the new first-line treatment for severe malaria [7, 14, 15]. However, drugs officially withdrawn from therapeutic practice have often persisted out of control in the market and are widely used against malaria, alongside drugs not promoted by national policy (e.g., mefloquine or piperaquine containing ACTs) [6, 9, 18].

Overall, a landscape conducive to the emergence and spread of resistance has taken shape along the history of the DRC’s national malaria policy. Artemisinin-resistant malaria, which appeared in the Greater Mekong subregion with the first clinical failures of ACTs, has recently emerged in countries bordering the DRC (e.g., Rwanda and Uganda) and raises serious concern for the country as for the whole African continent [20–24]. By accounting for over 10% of the worldwide malaria burden yearly, the country could thus potentially become a global hub for drug-resistant malaria [25]. This situation is most worrying especially as alternatives for replacing front-line drugs such as artemisinin derivatives remain very limited or would require several years to be developed and implemented [26]. This emphasizes the critical need for a national system to monitor and track drug-resistant malaria for global health perspectives. Therefore, this article initiates a living systematic review aiming at periodically summarizing the distribution of malaria parasites carrying molecular markers of drug resistance across the DRC to support customized public health decision-making and surveillance efforts.

## Methods

### Search strategy and resource identification

We conducted this systematic review following the PRISMA (“Preferred Reporting Items for Systematic Reviews and Meta-Analyses”) guidelines (Additional file 1: Table S1) [27, 28]. The review steps were independently performed by two groups of investigators (i.e., NKK/ARA and ETK/NK) and their results were cross-checked to reduce possible errors during the search of information sources and the integration of evidence retrieved from primary articles. Any discrepancies likely arising from the process were resolved by consensus. We searched seven databases (i.e., PubMed, Embase, Scopus, African Journal Online, African Index Medicus, Bioline, and Web of Science) for articles published before July 2023. These articles had to report on clinical *Plasmodium* isolates sampled within the DRC and that had been genotyped for the detection of molecular markers of drug resistance. The search strategy pre-defined for this purpose was built using English and French versions of specific keywords including the names of genes potentially encoding known molecular markers of drug resistance (Additional file 1: Table S2). No filter was applied to the literature search to ensure the widest inclusion of potentially informative resources. Bibliographic listings contained in previous articles were manually searched for additional articles to be eventually considered for the review.

### Selection criteria

We used pre-defined inclusion and exclusion criteria following a PICOS framework—i.e., Population, Intervention, Comparator, Outcomes, and Study designs—(Additional file 1: Table S3) to assess the eligibility of primary articles. Eligible articles were those reporting original observational data on molecular markers of drug resistance (genotype and frequency) in *Plasmodium* isolates collected from individuals residing in the DRC. When the data from a specific survey were used in subsequent publications, we captured only the most recent information in the inclusion process. Studies that focused on subjects traveling to or outside the country were excluded along with articles reporting insufficient information (e.g., unknown isolate frequency), systematic reviews, case reports, conference presentations, conference abstracts and correspondence to editors. We applied a modified version of the Newcastle–Ottawa Scale (NOS) to assess the quality of primary articles based on three criteria: the representativeness of the study samples (rated on a maximum of one star), the sample size (rated on a maximum of one star), and the result of the study (rated on a maximum of three stars) [29]. Only articles of methodological quality rated as moderate (two to three stars on the NOS) or high (four to five stars on the NOS) were considered for inclusion in this systematic review.

### Data collection and management

We carried out the data collection according to a sequential process (i.e., literature search, assessment and inclusion of resources, validation and extraction of data). We reviewed each study that met the selection criteria for extracting information related to study characteristics and to genotypes of drug resistance driving genes. Surveys from large geographic area (larger than a city, a town or a village) that could not be separated by sites were treated as of unknown location. Data that were not made available through primary articles were requested from corresponding authors.

### Data synthesis and risk of bias assessment

A narrative summary of the information collected was produced referring to absolute isolate numbers as well as median values and corresponding interquartile ranges (IQRs) for relative proportions of isolates carrying specific genotypes, such as copy number variations, wild-type genotypes, or single-nucleotide polymorphisms (SNPs). Whenever possible, we summarized haplotype variations for alleles jointly reported on codon-positions 72 to 76 of the *P. falciparum* chloroquine resistance transporter (*Pf*CRT), on codon-positions 51, 59, and 108 of the dihydrofolate reductase (*Pf*DHFR) as well as on codon-positions 437 and 540 of the dihydropteroate synthase (*Pf*DHPS). We linked each survey to its year (or midpoint year) of sampling and its geographic location to display spatial or temporal patterns of parasites potentially carrying different genotypes. The R software version 4.2.0 [30] was used to perform data analysis and mapping. The risk of bias was minimized by excluding traveling malaria cases as well as repeated communications on same isolates. In addition, the NOS criteria used to assess the methodological quality of the primary articles would have minimized the risks of selection bias, confounding factors, and performance bias in the studies considered for this review [31].

## Results

### Basic characteristics of primary studies

**Fig. 1:**
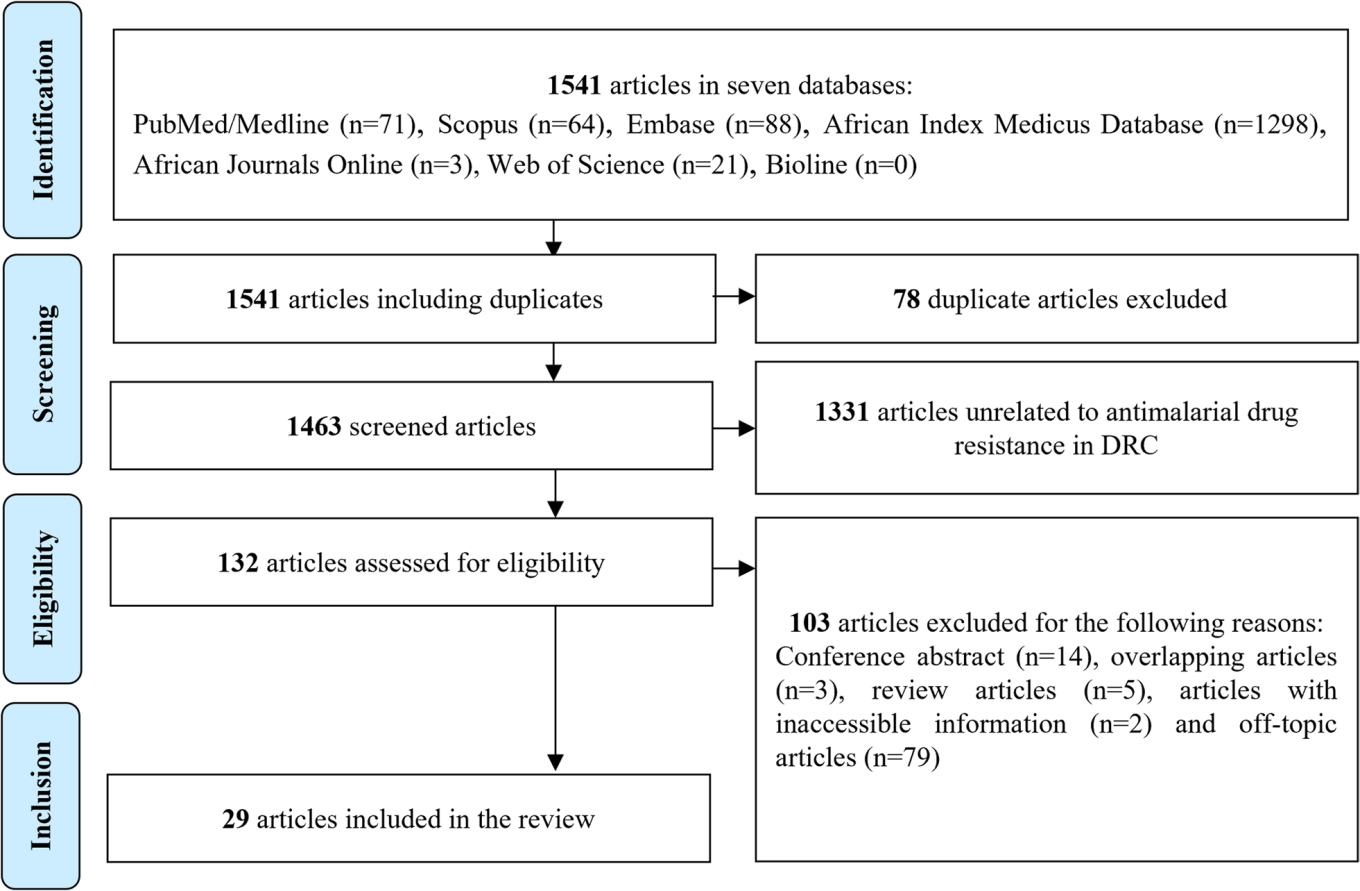
PRISMA diagram of the systematic review. This figure shows the steps followed by this systematic review according to the PRISMA (“Preferred Reporting Items for Systematic Reviews and Meta-Analyses”) guidelines. Overall, out of 1541 articles retrieved from seven databases, 29 were finally included in the data collection process

We aggregated 1541 articles found in different databases through the literature search strategy with no additional studies obtained by hand search. By excluding 78 duplicated articles, we screened 1463 publications of which 1434 were found to be ineligible based on criteria defined for this review (Fig. 1). Finally, we could include 29 articles in the review. These articles reported on 6385 *P. falciparum* specimens sampled between 2000 and 2020 from different sites and that had been successfully genotyped to determine potential drug-resistance molecular markers encoded in the following genes: *pfdhfr*, *pfdhps*, *pfcrt*, *pfk13*, *pfmdr1,* and *pfmdr2* (Additional file 1: Table S4). An overview of the antimalarial drug resistance landscape in the country is presented in Table 1. The distribution of surveys varied considerably over time and geographical space with most frequent molecular surveillance covering Kinshasa, the country's capital city (16 out of 26 studies of known location). We noted that the largest gaps in geographic coverage of surveillance were in areas in the north and center of the country (Additional file 1: Fig. S1).

**Table 1:** Summary of the landscape of drug-resistant malaria in the DRC as of June 2023

Antimalarial drug	Malaria drug-resistance in the DRC as of June 2023
Quinine	• Quinine-resistant malaria was not confirmed, since there is still no validated molecular marker; but it was only suspected given several isolates carrying *Pf*CRT K76T and *Pf*MDR-1 D1246Y mutations
Lumefantrine	• Lumefantrine-resistant malaria was suspected given isolates potentially carrying *Pf*MDR1 the NFD haplotype which consists of N86, Y184F, and D1246 (but there is still no know validated marker for this resistance)
Mefloquine	• Mefloquine-resistant malaria was not detected as no isolate was detected with amplified copy numbers of *pfmdr1* and *pfmdr2* genes
Chloroquine	• Median 32.4% [IQR: 45.6] of isolates were chloroquine resistant as they carried a *Pf*CRT K76T mutation predominately onto a background with CVIET haplotypes • *Pf*CRT K76T carriage by parasites substantially decreased from 2000 to 2020 • Wide geographic variations in the prevalence of *Pf*CRT K76T parasites, however, was persisting in 2020 (1.8 to 89.5%) with increased risks of rebound due to the massive reintroduction and misuse of chloroquine for putative treatment or prevention of COVID-19
Amodiaquine	• Amodiaquine-resistant malaria was not confirmed as no parasite isolate carried a *Pf*CRT SVMNT haplotype, but it was suspected, since up several isolates carried *Pf*CRT N86Y and D1246Y mutations (and, therefore, possibly encoded the YYY haplotype consisting of N86Y, Y184 and D1246Y)
Piperaquine	• Piperaquine-resistant malaria was not explored (i.e., corresponding *Pf*CRT mutations and gene amplification for *Pf*PM2 and *Pf*PM3 were not analyzed)
Artemisinin and derivatives	• Artemisinin-resistant malaria was not established as only a single isolate (sampled in 2013–2014) was detected with a R561H mutation that mediates for resistance. However, there is significant risk of local emergence or regional expansion of ART-resistant parasites from neighboring countries with reported emerging resistance (e.g., Uganda, Rwanda, and Tanzania) or from sites found with reduced levels of drug efficacy with ACTs • Isolates harboring mutations that structurally mimic known molecular markers of artemisinin resistance need to be monitored and investigated
Pyronaridine	• Pyronaridine-resistant malaria was not explored, since corresponding mutations of the *Pf*MRP1 were not analyzed
Proguanil	• The genetic background of the parasites suggests that proguanil-resistant malaria is very common (e.g., > 70% of parasites carry *Pf*DHFR S108N, N51I, and C59R), suggesting caution in the use of a chemoprophylaxis including PRO (e.g., PRO–AV combination) when traveling to the DRC
Sulfadoxine–Pyriméthamine (S–P)	• S–P-resistant malaria was widespread at high frequencies but with a moderate molecular profile (*Pf*DHPS A437G: 88.0% [IQR: 33.6]; *Pf*DHPS K540E: 38.9% [IQR: 47.7]) • Quintuple mutants (i.e., IRN–GE) were identified in 13.1% of parasites with highest prevalence in areas located in East parts of the country

### *Plasmodium falciparum* resistance to quinoline derivative drugs in the DRC

**Fig. 2:**
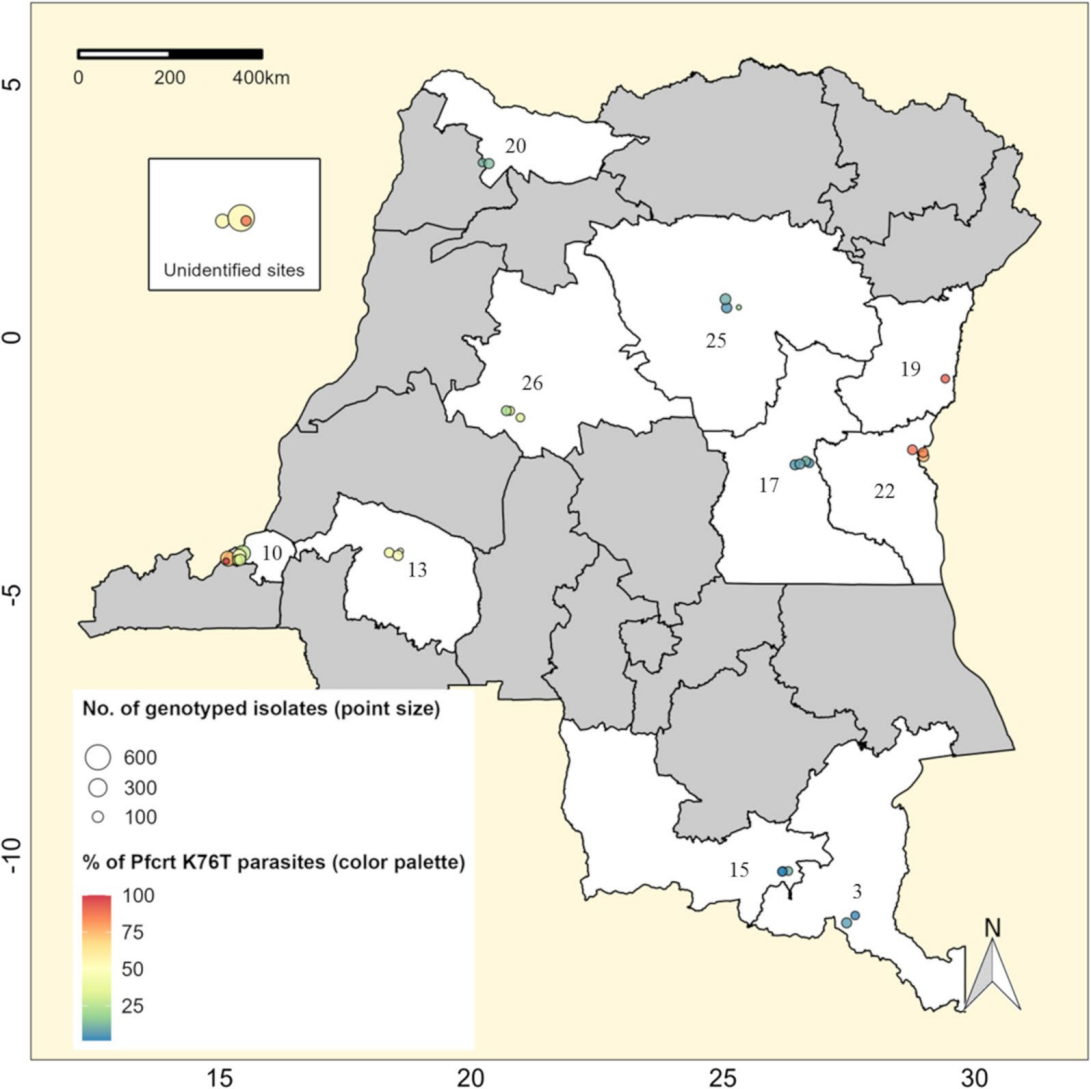
Distribution of malaria parasites encoding the *Pf*CRT K76T mutation in the DRC. This map displays single surveys that analyzed the *Pf*CRT K76T (a molecular marker of *P. falciparum* chloroquine resistance). Unshaded areas represent the country’s provinces, where parasites potentially carrying a *Pf*CRT K76T mutation have been surveyed (i.e., Haut-Katanga: 3; Kinshasa:10; Kwilu: 13; Lualaba: 15; Maniema: 17; Nord-Kivu: 19; Nord-Ubangi: 20; Sud-Kivu: 22; Tshopo: 25; and Tshuapa: 26). Circles represent surveys from different locations with a diameter proportional to the sample size of parasites that have been successfully genotyped on the codon-position likely encoding the *Pf*CRT K76T mutation. The color palette reflects the relative frequency of the *Pf*CRT K76T parasites during individual surveys

**Fig. 3:**
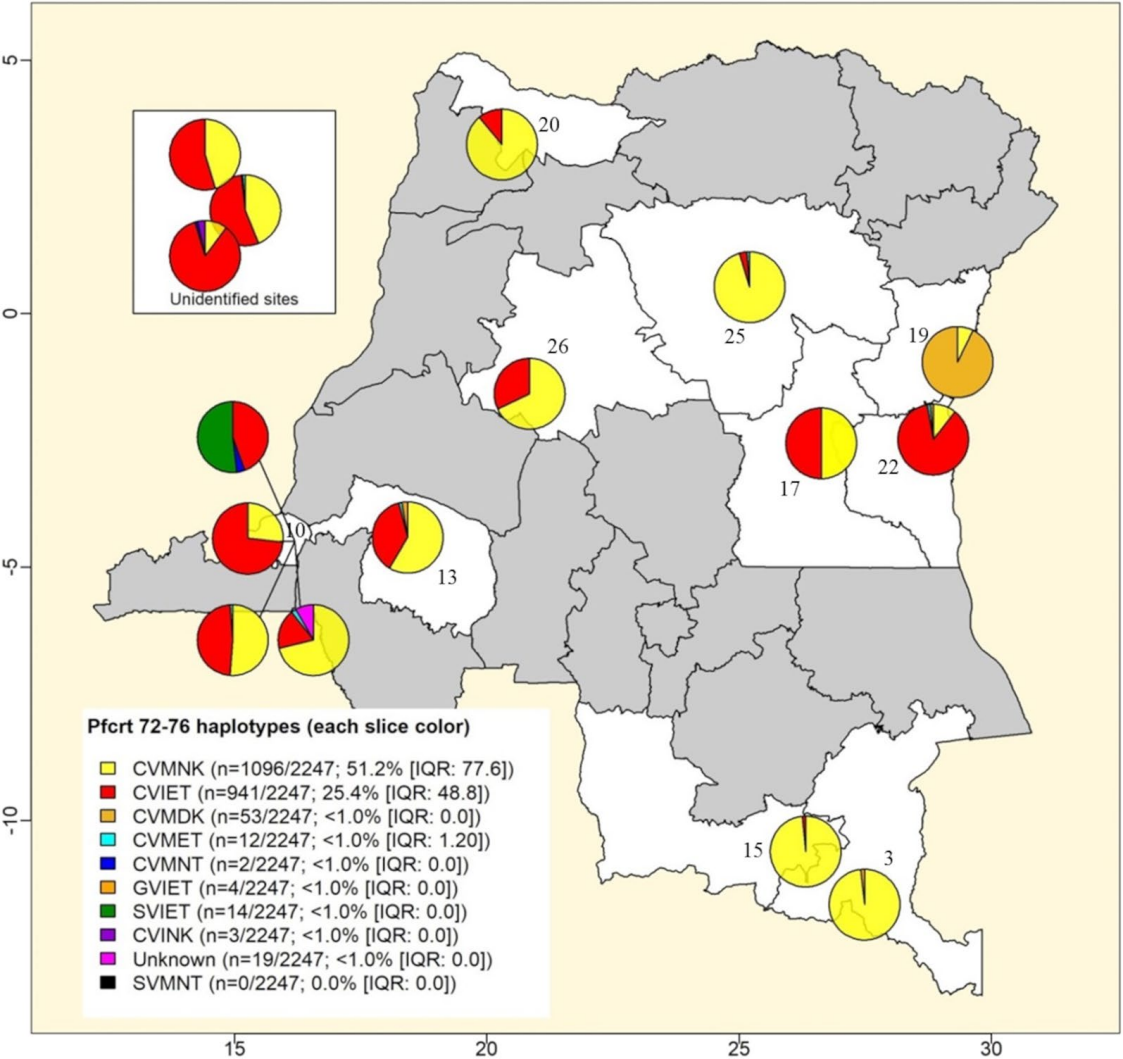
Distribution of malaria parasites encoding different *Pf*CRT haplotypes. This map displays single surveys that analyzed haplotype variations for alleles reported on *Pf*CRT 72–76 codon-positions. At least nine different *Pf*CRT 72–76 variations comprising mainly the wild-type CVMNK (i.e., C72–V73–M74–N75–K76) and the CVIET (i.e., C72–V73–M74I–N75E–K76T) haplotypes were recorded and are indicated by specific colors. Pie charts shown on the map represent proportions of isolates harboring each of the *Pf*CRT 72–76 haplotypes in individual surveys. Unshaded areas are provinces, where the *Pf*CRT 72–76 haplotypes was analyzed (i.e., Haut-Katanga: 3; Kinshasa:10; Kwilu: 13; Lualaba: 15; Maniema: 17; Nord-Kivu: 19; Nord-Ubangi: 20; Sud-Kivu: 22; Tshopo: 25; and Tshuapa: 26)

SNPs along two key transporter proteins, *Pf*CRT (encoded by the PF3D7_0709000 gene on chromosome 7) and “*Plasmodium falciparum* multidrug resistance 1” (*Pf*MDR1, encoded by the PF3D7_0523000 gene on chromosome 5), were first discovered to confer resistance to quinoline derivatives drugs in the 1990s [32]. Since then, the *Pf*CRT K76T mutation has emerged as the main molecular marker mediating the *P. falciparum* chloroquine-resistance [32]. This mutation was thus extensively sought in this review (11 articles conducted from 2000 to 2019 and including total 3464 isolates) and resulted in an overall median 32.4% [IQR: 45.6] frequency among parasites collected across different locations with highest frequencies found in eastern parts of the country (Fig. 2, Additional file 1: Fig. S2). We noted a decrease in the median proportion of isolates carrying this point mutation per study, from 100.0% [IQR: 0.0] in 2000 (i.e., all 27 isolates genotyped within a single study) to 13.3% [IQR: 23.2] in 2019 (i.e., median 13 out of 95 genotyped isolates per survey) (Additional file 1: Table S5), despite that wide geographical variations (e.g., 1.8–89.5%) were still found in most recent surveys [33, 34]. Consistently, *Pf*MDR1 SNPs involved in decreased parasite susceptibility to chloroquine were also frequently observed, including N86Y (52.6% [IQR: 28.7]), Y184F (43.8% [IQR: 8.8]), and D1246Y (23.3% [IQR: 36.4]) mutations (Additional file 1: Fig. S3) [35–37]. Interestingly, the *Pf*MDR1 N86Y, Y184, and D1246Y genotypes (i.e., the *Pf*MDR1 YYY haplotype) possibly occurring on a *Pf*CRT K76T genetic background suggest that parasites with reduced susceptibility to amodiaquine were likely circulating in the country [38, 39]. Likewise, the *Pf*MDR1 NFD haplotype (consisting of *Pf*MDR1 N86, Y184F, and D1246 genotypes) potentially associated with a wild-type *Pf*CRT 76 codon could possibly be harbored by circulating parasites with reduced susceptibility to lumefantrine [39]. Similarly, the data collected [35–37] provide insight into the possibility of parasites with reduced sensitivity to quinine due to the potential combination of *Pf*MDR1 D1246Y and *Pf*CRT K76T mutations [32] (Additional file 1: Fig. S3). To further explore the molecular profile of parasites carrying the *Pf*CRT K76T allele, we focused on different *Pf*CRT haplotypes resulting from amino acids variations on codon-positions 72 to 76 (Fig. 3). Therefore, we found that the *Pf*CRT CVIET (i.e., C72–V73–M74I–N75E–K76T) was the most frequent mutant haplotype (25.4% [IQR: 48.8]), whereas other mutant haplotypes were detected at very low median frequency per site (< 1% parasites, each). Overall, median 51.2% [IQR: 77.6] of parasites carried the wild-type *Pf*CRT CVMNK haplotype (i.e., C72–V73–M74–N75–K76). We spotted no parasite carrying a *Pf*CRT SVMNT haplotype (i.e., C72S–V73–M74I–N75–K76T), a well-established marker of amodiaquine resistance [40] (Fig. 3). Finally, we did not identify any parasite isolates encoding gene copy number variations for the “*Plasmodium falciparum* multidrug resistance 2” (*Pf*MDR2; n = 2 isolates) and the *Pf*MDR1 (n = 366 isolates) that would have suggested potential resistance of *P. falciparum* to mefloquine (Additional file 1: Fig. S4).

### *Plasmodium falciparum* resistance to artemisinin derivative drugs in the DRC

**Fig. 4:**
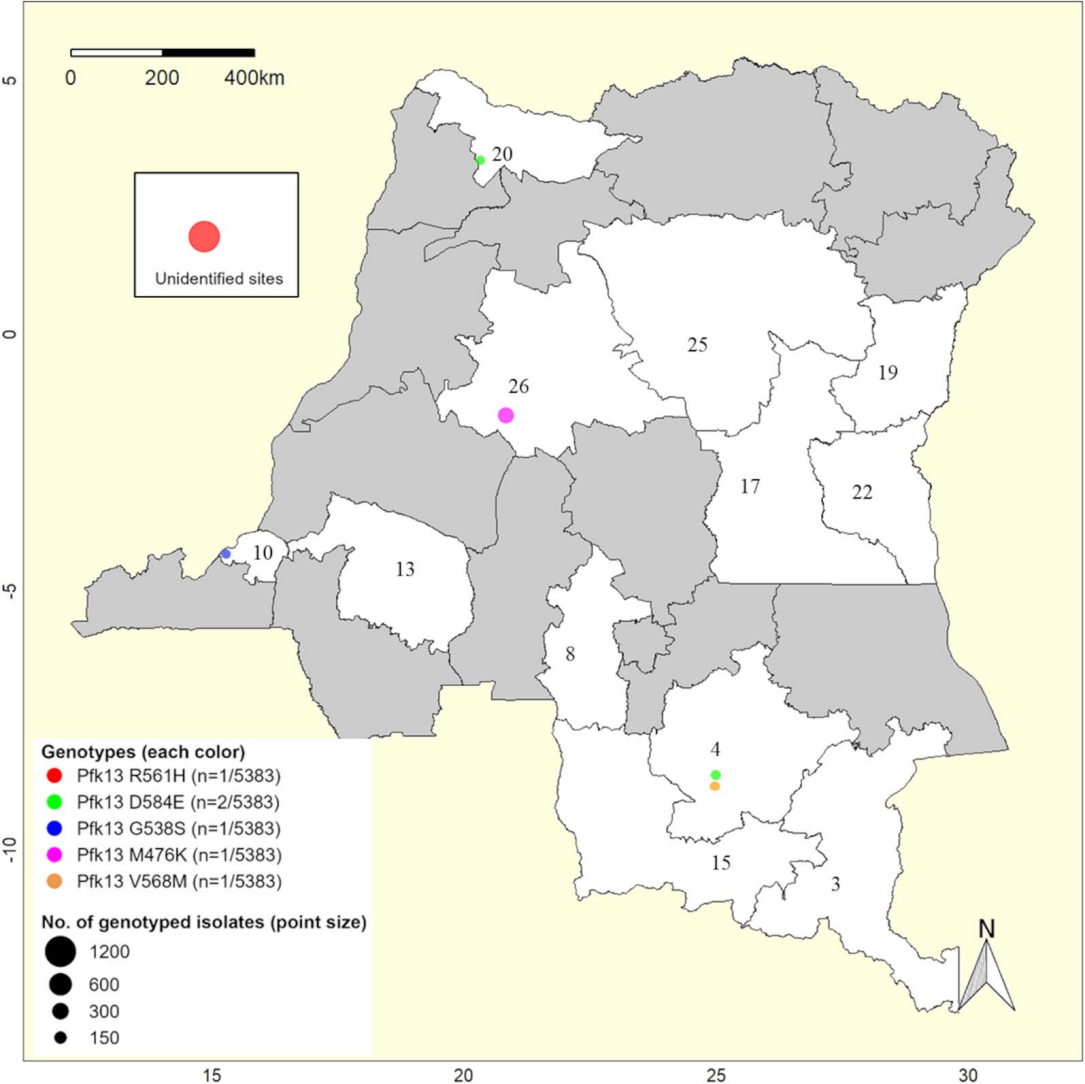
Distribution of *Plasmodium* parasites carrying *Pf*K13 mutations potentially linked to artemisinin (ART) resistance in the DRC. Each circle shown on this map reflects a survey that reported at least one malaria parasite carrying a mutation onto a *Pf*K13’s locus that is validated or suspected for driving ART resistance. The diameter of circles is proportional to the number of isolates for which the *Pf*K13 sequence has been successfully genotyped. Mutations are shown with absolute frequencies across different surveyed sites. Unshaded areas reflect provinces that have been monitored for parasites harboring *Pf*K13 mutations (i.e., Haut-Katanga: 3; Lualaba: 4; Kasai-Central: 8; Kinshasa:10; Kwilu: 13; Lualaba: 15; Maniema: 17; Nord-Kivu: 19; Nord-Ubangi: 20; Sud-Kivu: 22; Tshopo: 25; and Tshuapa: 26)

Several SNPs of a gene located on the chromosome 13 which encode the *P. falciparum* Kelch 13 protein (*Pf*K13) have been involved in resistance to artemisinin and its derivatives [41–43]. Through this review, eleven articles analyzed the genetic polymorphism of the *Pf*K13 in 5383 *P. falciparum* isolates collected from 2005 to 2019 (Additional file 1: Table S4). Therefore, a median frequency of 98.9% [IQR: 0.84] isolates displayed a conserved *Pf*K13 sequence (i.e., a *Pf*K13 of wild type or with only synonymous mutations), while 1.1% [IQR: 0.84] carried at least one non-synonymous mutation (Additional file 1: Fig. S5). Notably, out of 34 different non-synonymous mutations found in 78 isolates, 30 were located on the *Pf*K13 Propeller domain (i.e., above the codon-position 440 [44]) (Additional file 1: Table S6). Unlike all other surveys targeting the PfK13 Propeller domain, Miotto et al. [45] sequenced the full-length *Pf*K13 and were able to report four non-synonymous mutations located outside the Propeller domain (i.e., K92N, T149S, K189T and R225K) that are not associated with artemisinin resistance. We highlighted a set of five mutations recorded at relative frequencies < 1% and located on codon-positions that had been linked to artemisinin resistance in Southeast Asia (Fig. 4). Interestingly, among these mutations, we have recorded a single Congolese parasite with a *Pf*K13 R561H mutation which is known as a validated marker of artemisinin resistance in Southeast Asia and has been involved over the last 3 years in the emergence of drug-resistant parasites and clinical failures of ACTs in countries bordering the DRC, particularly in Tanzania, Uganda, and Rwanda [20, 22, 46, 47]. The remaining four mutations also warrant interest as they structurally mimic SNPs linked in vivo or in vitro to artemisinin resistance in Southeast Asia (i.e., M476K mimicking M476I, G538S mimicking G538V, V568M mimicking V568G and D584E mimicking D584V). We finally observed that all other *Pf*K13 mutations found in the country, except S522C [41], have not been explored clinically and experimentally to rule out any biological relevance.

### *Plasmodium falciparum* resistance to antifolate drugs in the DRC

**Fig. 5:**
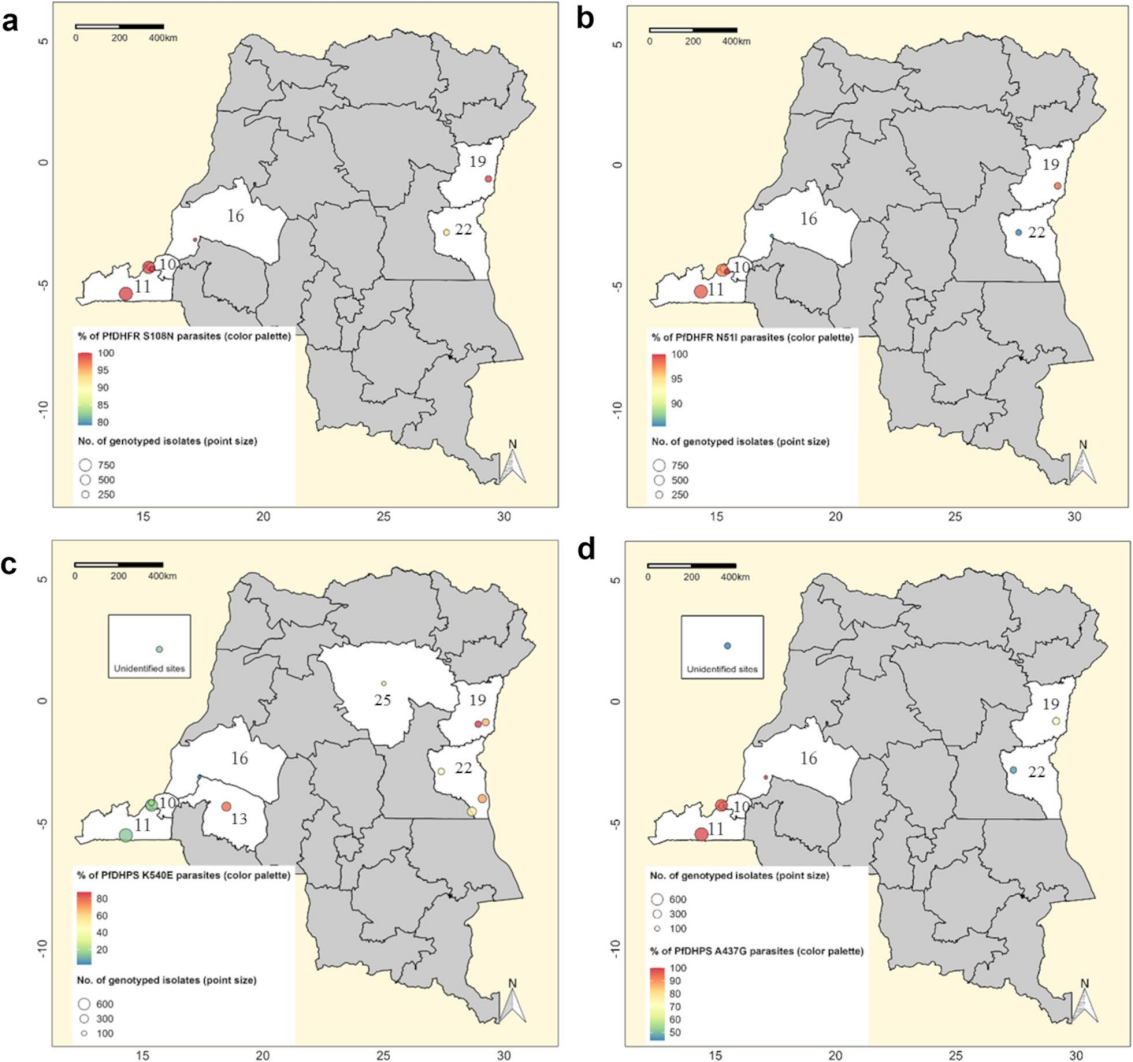
Distribution of *P. falciparum* parasites carrying major *Pf*DHFR and *Pf*DHPS mutations in the DRC. Surveys that analyzed each of *Pf*DHFR or *Pf*DHPS mutations were projected on these maps. The diameter of each circle is proportional to the number of isolates that have been successfully sequenced for corresponding genes. The color palettes reflect the relative frequency of parasites carrying each mutation during individual investigations. **a**–**d** display information related to *Pf*DHFR S108N, N51I, K504E, and A437G mutations separately. Unshaded areas thus represent provinces that report parasites harboring these mutations (i.e., Kinshasa: 10; Kongo-Central: 11; Mai-Ndombe: 16; Nord-Kivu: 19; Sud-Kivu: 22)

**Fig. 6:**
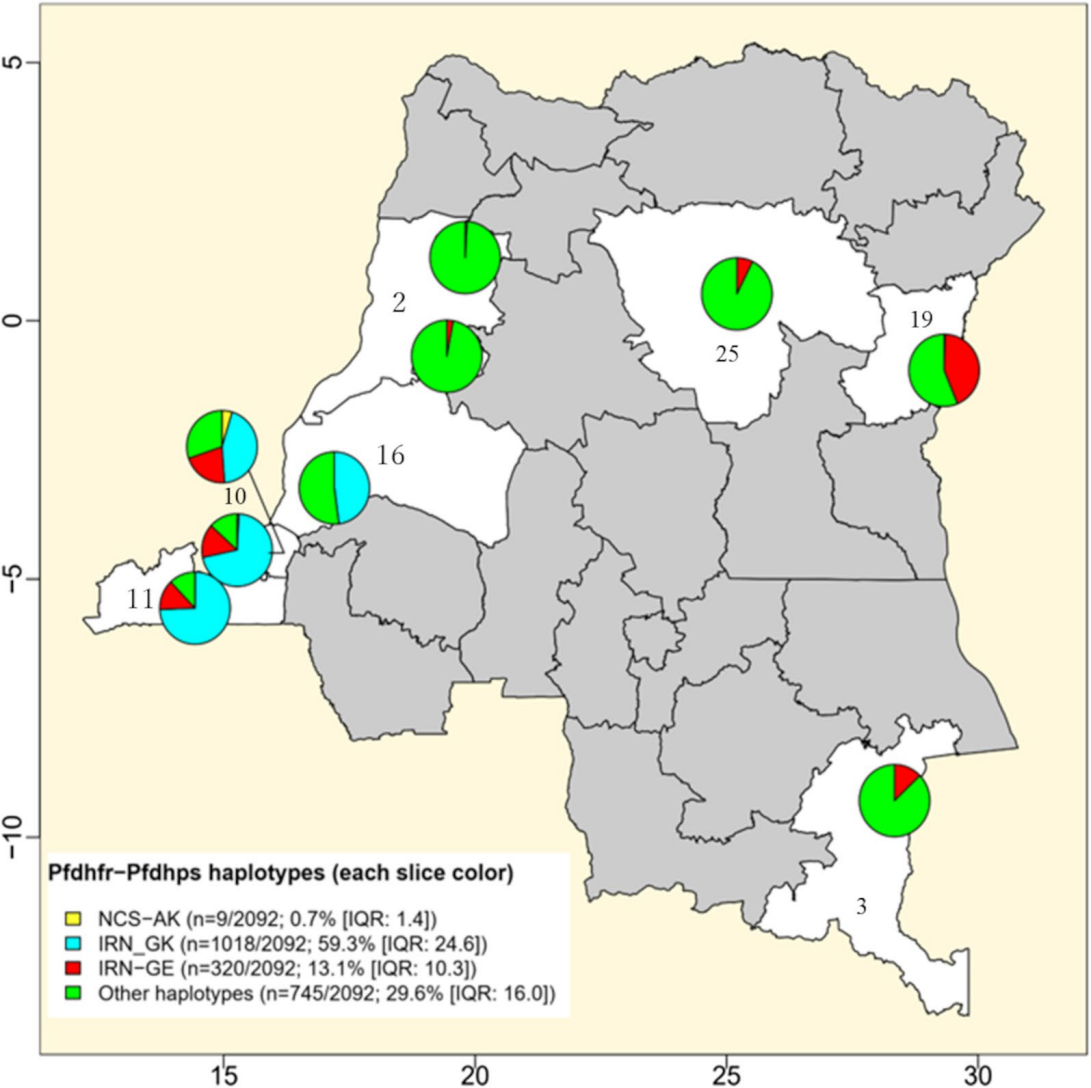
Distribution of malaria parasites harboring the quintuple mutant *Pf*DHFR–*Pf*DHPS haplotypes. This map displays each survey reporting major haplotype variations for alleles of *Pf*DHFR 51–59–108 and *Pf*DHPS 437–540 codon-positions. Pie charts shown on this map reflect proportions of isolates harboring the wild-type NCS–AK haplotype (i.e., *Pf*DHFR N51–C59–S108 and *Pf*DHPS A437–K540), the quadruple mutant IRN–GK (i.e., *Pf*DHFR N51I–C59R–S108N and *Pf*DHPS A437G–K540), the quintuple mutant IRN–GE (i.e., *Pf*DHFR N51I–C59R–S108N and *Pf*DHPS A437G–K540E), or other haplotypes within single surveys. Unshaded areas correspond to provinces that have been explored for parasites carrying different *Pf*DHFR–*Pf*DHPS haplotypes so far (i.e., Equateur: 2; Haut-Katanga: 3; Kinshasa:10; Kongo-Central: 11; Mai-Ndombe: 16; Nord-Kivu: 19; Sud-Kivu: 22)

Genetic mutations in genes encoding two enzymes, the *Pf*DHFR and the *Pf*DHPS, are known as conferring resistance of *P. falciparum* to antifolate drugs, namely, S–P since in the 1990s [48]. These mutations are thus widely explored for surveillance purposes [32]. In this review, we gathered total 3537 isolates (ten articles) and 3518 *P. falciparum* isolates (twelve articles) that have been, respectively, genotyped, from 2002 to 2020, for specific mutations of *Pf*DHFR (at any of the C50, N51, C59, S108, and I164 codon-positions) and *Pf*DHPS (at any of the I431, S436, A437, K540, A581, and A613 codon-positions) (Table 2). We thus found that most prevalent mutations were *Pf*DHFR S108N (99.5% [IQR: 3.9]) and N51I (97.9% [IQR: 25.0]) as well as *Pf*DHPS A437G (88.0% [IQR: 33.6]) and K540E (38.9% [IQR: 47.7]) (Fig. 5). *Pf*DHFR SNPs were ubiquitous across the country, while *Pf*DHPS ones predominated either in the western (for A437G) or in eastern parts (for K540E) of the country (Fig. 5). Furthermore, we assessed *Pf*DHFR–*Pf*DHPS haplotype combinations for 2098 isolates from five articles that jointly provided genetic polymorphism data on three *Pf*DHFR (i.e., N51, C59, and S108) and two *Pf*DHPS codon-positions (i.e., A437 and K540). We thus identified parasites harboring thirteen different *Pf*DHFR–*Pf*DHPS haplotypes (i.e., NCS–AK, ICN–AK, IRN–GE, ICN–GE, ICN–GK, IRN–AE, IRN–AK, IRN–GK, NCN–GK, NCS–GE, NCS–GK, NRN–AK, NRN–GK) (Additional file 1: Table S7). In absolute terms, the most frequent *Pf*DHFR–*Pf*DHPS haplotypes were quadruple IRN–GK mutants (59.3%; n = 1018) comprising three *Pf*DHFR mutations (N51I, C59R, and S108N) along with *Pf*DHPS A437G, followed by quintuple IRN–GE mutants that encoded an additional *Pf*DHPS K540E mutation (13.1%; n = 311). These mutants were most prevalent in eastern regions of the country (Fig. 6).

**Table 2:** Frequency of genetic alleles potentially linked to malaria resistance to anti-folate drugs in the DRC

Alleles	n*	m*	Median % of mutants (IQR)
*Pf*DHPS alleles			
I431V	1588	14	1.4 (0.9)
S436A	2165	297	7.4 (13.9)
A437G	2103	1821	88.0 (33.6)
K540E	3518	1373	38.9 (47.7)
A581G	3076	431	10.7 (17.7)
A613S	1684	10	0.1 (0.5)
*Pf*DHFR alleles			
N51I	2324	2260	97.9 (25.0)
C59R	2324	1937	79.1 (0.0625)
S108N	2324	2291	99.5 (11.9)
I164L	2927	2	< 0.1 (3.94)

(*) *n*: no. of genotyped isolates; m: no. of detected mutants

## Discussion

This review summarizes information from 6385 *P. falciparum* isolates sampled across the DRC over the past two decades and provides a baseline for enhanced country–level drug resistance surveillance efforts. Indeed, these parasites have been analyzed for genetic mutations that reflect antimalarial drug resistance with relevance for health policy [32]. Therefore, this work and subsequent updates through an intended living systematic review process [49] have the potential to support a continuous monitoring of drug-resistant malaria through the country while supporting evidence-based public health decision making and identifying surveillance gaps to be addressed. So far, resistance surveillance activities targeted drugs historically used against malaria in the country, including quinolines (i.e., quinine, chloroquine, amodiaquine, mefloquine, and lumefantrine), artemisinin derivatives, and antifolate drugs (i.e., S–P) [7, 50]. Overall, we detected malaria parasites displaying mutations reflecting or raising suspicion of resistance to all these drugs, except for mefloquine. However, the magnitude of detected resistance mostly warranted additional explorations given limited number of surveys, gaps in geographic coverage, and asymmetrical surveillance activities prioritizing Kinshasa, the country's capital. In this context, we advocate for the democratization of monitoring efforts to partially overcome existing disparities. Such efforts have become more achievable in resource-limited settings, such as most parts of the DRC, thanks to recent advances in portable, low-cost sequencing platforms that have gained momentum as an alternative to heavy central laboratories for the detection of antimalarial drug resistance markers [51–54]. Therefore, the prospect of coupling molecular surveillance with in vivo clinical trials and in vitro drug susceptibility testing becomes more conceivable and desired to adequately inform policies aimed at containing the emergence and spread of drug resistance antimalarial drugs in the country.

With respect to resistance to quinolone-containing antimalarial drugs, surveillance activities were dominated by monitoring *Pf*CRT K76T mutations that confer resistance to chloroquine but possibly contribute to reduced susceptibility to other drugs, such as quinine, amodiaquine and lumefantrine [32]. Consistently with outcomes from other Sub-Saharan regions [55], we found that the overall proportion of parasites carrying this mutation has decreased overtime in the DRC. This suggests a gradual recovery of chloroquine susceptibility among malaria parasites following the lifting of the drug selective pressure after its withdrawal from clinical use since 2002 [13]. However, due to residual locations persisting at very high proportions of resistant parasites [33, 34], the frequency of *Pf*CRT K76T parasites remained very heterogeneous in this review. From a policy standpoint, this geographic heterogeneity of the distribution of chloroquine susceptible parasites has important implications. First, the average > 30% of *Pf*CRT K76T parasites (which is higher than the 10% threshold set by the WHO for enacting a drug policy change [56]) prevents any short-term reintroduction of the drug into clinical practice in the country. Residual locations with higher chloroquine resistance could be due to a local fixation of the *Pf*CRT K76T mutation prior to chloroquine withdrawal, raising uncertainties around a full recovery of the susceptibility of parasites to chloroquine in the future. In addition, the widespread use of amodiaquine as part of first-line ACTs could be sustaining K76T parasites, since the drug pressure can *Pf*CRT CVIET haplotypes that carry a K76T mutation [32]. It is also likely that this resistance could be maintained due to persistent chloroquine use in the population at odds with national policies, as reported in other sub-Saharan African countries [57]. Further explorations and health policies accounting for within-country geographical variations are, therefore, needed [34]. Regulatory efforts to control the use of antimalarial drugs remain also relevant, especially since the ongoing the coronavirus disease 2019 (COVID-19) pandemic brought back to the fore the widespread use of chloroquine (and its derivative, hydroxy-chloroquine) raising fears of further drug-resistance development [58, 59]. Unlike widespread chloroquine resistance, no evidence suggesting any mefloquine resistance could be obtained while resistance to quinine, amodiaquine, and lumefantrine could only be suspected. However, these outcomes raise some cautions given limited evidence gathered in this review. First, these suspicions were based on a combination of specific *Pf*MDR1 and *Pf*CRT genotypes which still require causality validation through experimental studies [32, 38, 39]. Then, data contrasting with any lumefantrine or amodiaquine resistance were also obtained, including the absence of parasites encoding *Pf*CRT SVMNT haplotype [40] or *Pf*MDR1 S1034C and N1042D mutations [32]. Finally, the magnitude of possible resistance to quinolines other than chloroquine could not be captured across the country as, so far, only limited studies tracked *Pf*MDR1 SNPs [35, 36] and related haplotype combinations [37]. Likewise, resistance to piperaquine—encoded by additional *Pf*CRT SNPs [60, 61] as well as Plasmepsins 2 and 3 [62]—was not covered so far by surveillance activities. Therefore, while continuously monitoring chloroquine resistance is needed, further investigations and surveillance efforts are warranted to clear suspicions upon resistance to other quinoline compounds [9].

Furthermore, only a single malaria isolate sampled between 2013 and 2014 was carrying a mutation (i.e., *Pf*K13 R561H) listed among molecular markers validated for artemisinin resistance, has been recorded so far in this review [41]. It is thus unlikely that resistance to artemisinin derivatives is already established in the country. Nevertheless, this observation suggests that malaria parasites resistant to artemisinin could be circulating for a while at low frequencies in the Congolese population, raising fears of their possible selection and emergence under the pressure of current first-line treatments (i.e., ACTs and injectable artesunate, respectively, for uncomplicated and severe malaria). In addition, while emerging artemisinin-resistance driven by R561H, A675V, and C469Y mutations has been spreading in neighboring countries (i.e., Rwanda, Uganda, and Tanzania) during the last 3 years [41, 44, 46, 47, 63], alarming evidence of declining efficacy of ACTs have been recorded in Mikalayi, a town in the Kasai-Central region in the middle of the country [37]. Noteworthy, this efficacy of ACTs significantly decreased below the 90%-cutoff recommended by the WHO to consider a drug policy change [43] and has been also reported in an area located in Angola, not far from Mikalayi in DRC [64]. It is difficult to speculate on a possible link between the Congolese *Pf*K13 R561H parasite and the subsequent emergence of resistant malaria in the Great Lakes region, because its precise sampling site or time remains unknown and no further warning signs such as the selection of new mutant parasites could be reported across the DRC. However, all these events suggest that the DRC may be on the cusp of an epidemiological shift in the malaria landscape and should prompt health policymakers to undertake proactive measures to counter any possible emerging artemisinin resistance in the country. Therefore, the NMCP has considered introducing the artesunate–pyronaridine combination among first-line policies, alternatively to currently used ACTs—i.e., artesunate–amodiaquine and artemether–lumefantrine [16]. Diversifying first-line treatments would be beneficial from an epidemiological perspective as it can decrease the selective pressure and delay the emergence of artemisinin resistance and its spread across the population. Especially, pyronaridine would offer additional advantages as it has recognized resilience against the development of resistance, in addition to being less prone to cross-resistance with other antimalarial drugs [65] and having not yet been used in the country in the past [16]. Henceforth, artemisinin-based triple therapies (TACTs)—i.e., combinations of artemisinin with two partner drugs—could also be considered as an option [66, 67]. However, beyond the diversification of first-line treatment policies, we draw attention to the urgent need for the NMCP to undertake additional public health measures that can further delay the emergence and spread of artemisinin resistance and treatment failure while extending the therapeutic life of available drugs and improving the chances of eliminating malaria. Routine monitoring of molecular markers of resistance can provide crucial information on the spatial extent and evolutionary dynamics of resistant malaria to guide timely health decisions. This has proven to be practical and feasible in resource-limited settings, both at national and local scales in targeted regions [53, 54]. To this end, beside well-known *Pf*K13 Propeller domain mutations that drive artemisinin resistance in Southeast Asia [43, 44], surveillance platforms need to be flexible enough to include a broader set of newer molecular markers. In fact, cases of clinical failure with ACTs did not present the PfK13 Propeller domain mutations dedicated by the WHO to epidemiological surveillance, suggesting that other genetic loci might be locally involved in drug resistance [37]. It is obvious that African malaria parasites could use their specific genetic background to generate new resistance mechanisms outside the *Pf*K13 Propeller domain [68]. Hence, additional genetic loci of interest could include the *Pf*K13 ‘Broad-Complex Tramtrack and Bric a brac’ or ‘Poxvirus and Zinc finger’ domains (BTB/POZ) [69] as well as other loci, such as the *P. falciparum* Coronin gene [70]. In the same momentum, we had highlighted also the need for monitoring African parasites carrying *Pf*K13 SNPs that mimic well-known drug resistance markers, of which a few sporadic cases were observed in this review [44]. Additional research via whole genome sequencing efforts is also needed to validate these markers or even identify new ones. Either way, in vivo clinical trials monitoring the efficacy of artemisinin-based therapies should not be overlooked and should be continued, since they are decisive for any change in antimalarial therapeutic policy [71]. Furthermore, additional public health efforts are now required to further reduce the drug selective pressure upon the country and particularly in areas at high risk for the artemisinin resistance development, such as the Kasai-Central region and areas bordering Uganda, Rwanda, and Tanzania [25, 71]. Mass information campaigns and other public health measures aimed at limiting suboptimal absorption of artemisinin in the population (e.g., use of artemisinin monotherapies, consumption of *Artemisia* spp. plants, use of medications at sublethal doses due to incomplete prescription, partial intake, chemical alteration, or even drug counterfeiting) must, therefore, be considered in parallel with activities monitoring drug resistant parasites in migrants [44, 71].

Regarding malaria resistance to antifolate drugs, we found that despite widespread resistance to S–P across the DRC, the drug combination still retains some usefulness for malaria chemoprevention. Beyond the IPT currently implemented in the country during pregnancy, several WHO-recommended SP-based malaria chemoprevention strategies are, therefore, within reach, including perennial malaria chemoprevention for young children aged 12 at 24 months, seasonal malaria chemoprevention for children 3–59 months, and IPT in school-aged children 5–15 years [72]. Obviously, the molecular profile of this drug resistance corresponds to a moderate level of effectiveness for IPT in pregnancy, as per the van Eijk et al.’s definition criteria (i.e., *Pf*DHPS A437G ≥ 90% or *Pf*DHPS K540E ≥ 30% and < 90%) [73]. This implies that S–P may still be effective for preventing adverse pregnancy and birth outcomes (e.g., low birth weight, anemia) in the country more likely due to its additional non-malarial effects (e.g., antibiotic and immunomodulatory effects) [73–75]. However, the expected prophylactic effects of S–P against malarial infections may already have been lost; mother and fetus could, therefore, remain exposed to infection despite taking S–P [75, 76]. Moreover, considering that nearly 40% of parasites carried the *Pf*DHPS K540E substitution, S–P-based chemoprevention in children would still be indicated with respect to the cutoff criteria recommended by WHO (< 50% of *Pf*DHPS K540E parasites) [77]. The NMCP has thus already planned to implement S–P-based chemoprevention interventions in Congolese children [17]. Despite the perceived usefulness of these interventions, further implementation of S–P-based chemoprevention raises some concerns that should be brought to the attention of DRC health authorities. First, the risk of further selecting *Pf*DHPS K540E parasites and quintuple IRN–GE mutants should be managed properly and closely monitored to avoid rapidly reaching higher resistance levels and complete loss of the clinical efficacy of the drug [73, 78–80]. Second, combining S–P with amodiaquine, which has shown its effectiveness in the Sahel subregion of Africa [81], should be considered instead of simply the S–P combination. Finally, given the regional genetic background, local evidence (e.g., provided by clinical trials) of the prophylactic efficacy and the sustainability of any S–P-based strategy is needed [72, 78, 82]. As for chloroquine resistance, within-country variations and evolution dynamics in resistance profiles should anyway be taken in account when up scaling any S–P-based strategy in either pregnant women or children [79]. In particular, the higher prevalence of RN–GE parasites found in the eastern parts of the country should be regarded as local barriers to S–P-based policies that warrant alternative strategies [73, 83–86]. Furthermore, the molecular profile of the parasites (i.e., 99.5%, 97.9% and 79.1% of the parasites encoding the *Pf*DHFR mutations S108N, N51I and C59R, respectively) is also suggestive of frequent resistance to Proguanil, a drug antifolate widely used in combination with Atavaquone for chemoprophylaxis of malaria in travellers [87]. People visiting the DRC must, therefore, be warned of the serious threat that circulating resistant parasite could pose to the effectiveness of this malaria prevention strategy.

Overall, this systematic review had several limitations, including a limited number of primary articles, gaps in geographic coverage of monitoring activities, and high methodological heterogeneity in primary studies. Genetic markers of drug resistance were presented unrelated to information from in vivo assays and in vitro studies which would have further enriched this review by providing the maximum information on the emergence and evolution of drug resistant malaria in the population [44, 88, 89]. The scarcity of existing in vivo and in vitro studies is probably due to high costs and technical requirements. All these limitations restricted this work to a narrative review; but with desired progress in national malaria resistance surveillance efforts, in the future we hope to be able to update and report this review as an improved meta-analysis that addresses these weaknesses.

## Conclusions

Despite its shortcomings, this review highlights drug-resistant malaria as a major health problem and provides a basis for future surveillance efforts to guide public health efforts tailored to the country’s situation. Indeed, resistance to chloroquine remains high, resistance to sufadoxine–pyrimethamine undermines current chemoprevention strategies, while possible emergence of resistance to artemisinin threatens the country responsible for one-tenth of the world's malaria burden. Hopefully, the living systematic review launched with the current work will offer an approach to keep the high-quality evidence synthesis continuously up to date with most relevant and reliable information on drug resistance that can be used to inform policy and practice, and to ultimately improve quality of care and population health outcomes within the DRC and beyond.

### Supplementary Information

**Additional file 1: Table S1.** PRISMA checklist for the review of malaria drug resistance in the Democratic Republic of Congo. **Table S2.** Strategy used to search for articles published and included in the study. **Table S3.** PICOS criteria for the selection of primary articles published before July 2023. **Table S4.** Basic characteristics of articles included in this review article. **Table S5.** Evolution of the frequency of parasite carrying a *Pf*CRT K76T mutation in the DRC. **Table S6.** Overall proportions of parasites carrying non-synonymous mutations of the *Pf*K13 in the DRC. **Table S7.** Overall proportions of parasites encoding different *Pfdhfr*–*Pfdhps* haplotypes in the DRC. **Figure S1.** Geographic locations of collection sites in primary studies. **Figure S2.** Distribution of parasites carrying different *Pf*CRT mutations. **Figure S3.** Distribution of parasites carrying *Pfmdr*1 SNPs in the DRC. **Figure S4.** Distribution of parasites genotyped for *Pfmdr*1 and *Pfmdr2* gene copy number alterations in the DRC. **Figure S5.** Distribution of *Plasmodium* parasites carrying non-synonymous *Pf*K13 mutations in the DRC

## Data Availability

The data sets used and/or analysed during the current study are available from the corresponding author on reasonable request. The status of the subsequent living systematic review can be accessed at: https://nadinekayiba.pro/research/.

## Abbreviations

ACTs: Artemisinin-based combination therapies
BTB/POZ: The ‘Broad-Complex Tramtrack and Bric a brac’ or ‘Poxvirus and Zinc finger’ domains
COVID-19: Coronavirus disease 2019
DRC: Democratic Republic of Congo
IPT: Intermittent preventive treatment of malaria
NMCP: National Malaria Control Program
NOS: Newcastle–Ottawa Scale
PEV/LMTE: "*Programme Elargie de Vaccinationation et de Lutte contre les Maladies Transmissible de l’Enfance*"
PICOS: The framework Population, Intervention, Comparator, Outcomes, and Study designs
*Pf*CRT: The *P. falciparum* chloroquine resistance transporter
*Pf*DHFR: The *P. falciparum* dihydrofolate reductase
*Pf*DHPS: The *P. falciparum* dihydropteroate synthase
*Pf*K13: The *P. falciparum* Kelch 13 protein
*Pf*MRP1: The *Plasmodium falciparum* multidrug-associated resistance protein 1
*Pf*MRP2: The *Plasmodium falciparum* multidrug-associated resistance protein 2
PRISMA: Preferred Reporting Items for Systematic Reviews and Meta-Analyses
SNPs: Single-nucleotide polymorphisms
S–P: Sulfadoxine–pyrimethamine
USAID: United States Agency for International Development
WHO: World Health Organization
