# Supplementary material for: The landscape of drug resistance in Plasmodium falciparum malaria in the Democratic Republic of Congo: a mapping systematic review
Source: Trop Med Health. 2023 Nov 15;51:64. doi: 10.1186/s41182-023-00551-7 (PMC10647042; doi:10.1186/s41182-023-00551-7)
Supplement: Supplementary file 1 — Additional file 1: Table S1. PRISMA checklist for the review of malaria drug resistance in the Democratic Republic of Congo. Table S2. Strategy used to search for articles published and included in the study. Table S3. PICOS criteria for the selection of primary articles published before July 2023. Table S4. Basic characteristics of articles included in this review article. Table S5. Evolution of the frequency of parasite carrying a PfCRT K76T mutation in the DRC. Table S6. Overall proportions of parasites carrying non-synonymous mutations of the PfK13 in the DRC. Table S7. Overall proportions of parasites encoding different Pfdhfr–Pfdhps haplotypes in the DRC. Figure S1. Geographic locations of collection sites in primary studies. Figure S2. Distribution of parasites carrying different PfCRT mutations. Figure S3. Distribution of parasites carrying Pfmdr1 SNPs in the DRC. Figure S4. Distribution of parasites genotyped for Pfmdr1 and Pfmdr2 gene copy number alterations in the DRC. Figure S5. Distribution of Plasmodium parasites carrying non-synonymous PfK13 mutations in the DRC [file 41182_2023_551_MOESM1_ESM.pdf]

## Additional file

# The landscape of drug resistance in *Plasmodium falciparum* malaria in the Democratic Republic of Congo: a mapping systematic review

Nadine Kayiba Kalenda, Evariste Tshibangu-Kabamba, Angel Rosas-Aguirre, Natsuko Kaku, Yu Nakagama, Akira Kaneko, Dieudonné Mvumbi Makaba, Doudou Yobi Malekita, Brecht Devleesschauwer, Joris Losimba Likwela, Pius Kabututu Zakayi, Patrick DeMol, Georges Mvumbi Lelo, Marie-Pierre Hayette, Paul Lusamba Dikassa, Yasutoshi Kido[#], Niko Speybroeck.

[#]Correspondence:
Prof Yasutoshi Kido
Department of Virology and Parasitology & the Research Center for Infectious Disease Science (RCIDS), Osaka Metropolitan University
1-4-3 Asahi-cho, Abeno-ku, Osaka-shi, Osaka 545-8585 - Japan
kidoyasu@omu.ac.jp

### Contents

Contents ........ 1
Table S1: PRISMA checklist for the review of malaria drug resistance in the Democratic Republic of Congo. ... 2
Table S2. Strategy used to search for articles published and included in the study. ........ 4
Table S3. PICOS criteria for the selection of primary articles published before July 2023. ........ 4
Table S4. Basic characteristics of articles included in this review article. ........ 5
Figure S1. Geographic locations of collection sites in primary studies. ........ 6
Figure S2. Distribution of parasites carrying different *Pf*CRT mutations. ........ 7
Table S5. Evolution of the frequency of parasite carrying a *Pf*CRT K76T mutation in the DRC. ........ 8
Figure S3. Distribution of parasites carrying *Pfmdr*1 SNPs in the DRC. ........ 9
Figure S4. Distribution of parasites genotyped for *Pfmdr*1 and *Pfmdr2* gene copy number alterations in the DRC. ........ 10
Table S6. Overall proportions of parasites carrying non-synonymous mutations of the *Pf*K13 in the DRC ........ 11
Figure S5 Distribution of *Plasmodium* parasites carrying non-synonymous *Pf*K13 mutations in the DRC. ....... 12
Table S7. Overall proportions of parasites encoding different *Pfdhfr-Pfdhps* haplotypes in the DRC ........ 13
References ........ 14

**Table S1: PRISMA checklist [1] for the review of malaria drug resistance in the Democratic Republic of Congo.**

| Section/topic | # | Checklist item | Reported on page # |
|---|---|---|---|
| **Title** | | | |
| Title | 1 | Identify the report as a systematic review, meta-analysis, or both. | 1 |
| **Abstract** | | | |
| Structured summary | 2 | Provide a structured summary including, as applicable: background; objectives; data sources; study eligibility criteria, participants, and interventions; study appraisal and synthesis methods; results; limitations; conclusions and implications of key findings; systematic review registration number. | 2 |
| **Introduction** | | | |
| Rationale | 3 | Describe the rationale for the review in the context of what is already known. | 3-4 |
| Objectives | 4 | Provide an explicit statement of questions being addressed with reference to participants, interventions, comparisons, outcomes, and study design (PICOS). | 4 |
| **Methods** | | | |
| Protocol and registration | 5 | Indicate if a review protocol exists, if and where it can be accessed (e.g., Web address), and, if available, provide registration information including registration number. | - |
| Eligibility criteria | 6 | Specify study characteristics (e.g., PICOS, length of follow-up) and report characteristics (e.g., years considered, language, publication status) used as criteria for eligibility, giving rationale. | 5; Additional file p.4 |
| Information sources | 7 | Describe all information sources (e.g., databases with dates of coverage, contact with study authors to identify additional studies) in the search and date last searched. | 4-5 |
| Search | 8 | Present full electronic search strategy for at least one database, including any limits used, such that it could be repeated. | 4-5; Additional file p.4 |
| Study selection | 9 | State the process for selecting studies (i.e., screening, eligibility, included in systematic review, and, if applicable, included in the meta-analysis). | 5 |
| Data collection process | 10 | Describe method of data extraction from reports (e.g., piloted forms, independently, in duplicate) and any processes for obtaining and confirming data from investigators. | 5-6 |
| Data items | 11 | List and define all variables for which data were sought (e.g., PICOS, funding sources) and any assumptions and simplifications made. | 5-6; Additional file p.4 |
| Risk of bias in individual studies | 12 | Describe methods used for assessing risk of bias of individual studies (including specification of whether this was done at the study or outcome level), and how this information is to be used in any data synthesis. | 6 |
| Summary measures | 13 | State the principal summary measures (e.g., risk ratio, difference in means). | 6 |

| | | | |
|---|---|---|---|
| Synthesis of results | 14 | Describe the methods of handling data and combining results of studies, if done, including measures of consistency (e.g., $I^2$) for each meta-analysis. | 6-7 |
| Risk of bias across studies | 15 | Specify any assessment of risk of bias that may affect the cumulative evidence (e.g., publication bias, selective reporting within studies). | 7 |
| Additional analyses | 16 | Describe methods of additional analyses (e.g., sensitivity or subgroup analyses, meta-regression), if done, indicating which were pre-specified. | - |
| **Results** | | | |
| Study selection | 17 | Give numbers of studies screened, assessed for eligibility, and included in the review, with reasons for exclusions at each stage, ideally with a flow diagram. | 6-7 |
| Study characteristics | 18 | For each study, present characteristics for which data were extracted (e.g., study size, PICOS, follow-up period) and provide the citations. | 6-7 |
| Risk of bias within studies | 19 | Present data on risk of bias of each study and, if available, any outcome level assessment. | Additional file p.5 |
| Results of individual studies | 20 | For all outcomes considered (benefits or harms), present for each study: (a) simple summary data for each intervention group (b) effect estimates and confidence intervals, ideally with a forest plot. | Additional file p.5 |
| Synthesis of results | 21 | Present results of each meta-analysis done, including confidence intervals and measures of consistency. | 7-10 |
| Risk of bias across studies | 22 | Present results of any assessment of risk of bias across studies. | Additional file p.5 |
| Additional analysis | 23 | Give results of additional analyses, if done (e.g., sensitivity or subgroup analyses, meta-regression [see Item 16]). | - |
| **Discussion** | | | |
| Summary of evidence | 24 | Summarise the main findings including the strength of evidence for each main outcome; consider their relevance to key groups (e.g., healthcare providers, users, and policy makers). | 10-15 |
| Limitations | 25 | Discuss limitations at study and outcome level (e.g., risk of bias), and at review-level (e.g., incomplete retrieval of identified research, reporting bias). | 15 |
| Conclusions | 26 | Provide a general interpretation of the results in the context of other evidence, and implications for future research. | 15 |
| **Funding** | | | |
| Funding | 27 | Describe sources of funding for the systematic review and other support (e.g., supply of data); role of funders for the systematic review. | 17 |

**Table S2. Strategy used to search for articles published and included in the study.**

| | |
|---|---|
| #1 | (Malaria OR paludisme OR *Plasmodium*) |
| #2 | (“molecular marker” OR “marqueur moléculaire” OR *mdr1* OR *Pfmdr1* OR *Pfmdr2* OR *crt* OR *Pfcrt* OR *kelch13* OR *Pfkelch13* OR K13 OR *dhfr* OR *Pfdhfr* OR *dhps* OR *Pfdhps* OR *Pfpm3* OR *pm3* OR *Pfpm2* OR *pm2* OR *Pfcytb* OR *cytb* OR *Pfnhe1* OR *nhe1*) AND (resistance OR résistance OR resistant OR résistant) |
| #3 | (“Democratic Republic of Congo” OR “République Démocratique du Congo” OR Zaïre OR Congo) |
| Search | #1 AND #2 AND #3 |

**Table S3. PICOS criteria for the selection of primary articles published before July 2023.**

| **Parameters** | **Inclusion criteria** | **Exclusion criteria** |
|---|---|---|
| Population | • Individuals residing in the DRC and having been infected with malaria parasites. | • Traveling malaria cases detected outside the DRC. |
| Intervention | • Detection of *Plasmodium* parasites by PCR technique on blood samples with genotyping of any genes putatively involved in antimalarial drug resistance (i.e., *kelch13*, *mdr1*, *mdr2*, *crt*, *dhfr*, *dhps*, *pm3*, *pm2*, *cytb* and *nhe1*). | • No report on genes involved in antimalarial drug resistance |
| Comparator | • Criteria not applicable for this review. | • Criteria not applicable for this review |
| Outcomes | • Genotype of at least one locus on one of the following genes: *kelch13*, *mdr1*, *mdr2*, *crt*, *dhfr*, *dhps*, *pm3*, *pm2*, *cytb* and *nhe1* | • No reported data on genotypes and number of genotyped isolates |
| Study Design | • Peer-reviewed observational or clinical trial articles reporting on newly analyzed *Plasmodium* isolates | • Letters, commentaries, conference summaries, conference presentations;<br>• Systematic reviews and meta-analyzes;<br>• Studies based exclusively on animal models;<br>• Data relating to *Plasmodium* isolates genetically modified during laboratory experiments. |

**Table S4. Basic characteristics of articles included in this review article.**

| N° | Article | Data collection period | No. of sites | No. of samples | Analyzed molecular marker of drug resistance | Quality level |
|---|---|---|---|---|---|---|
| 1 | Alker AP et al. 2008 [2] | 2002 | 1 | 249 | *Pf*DHFR, *Pf*DHPS | Moderate |
| 2 | Antonia AL et al. 2014 [3] | 2007 | Unkown | 180 | *Pf*CRT | Moderate |
| 3 | Baraka V et al. 2017 [4] | 2012-2014 | 1 | 37 | *Pf*DHPS | Moderate |
| 4 | Baraka V et al. 2018 [5] | 2012-2014 | 1 | 2265 | *Pf*MDR1 | Moderate |
| 5 | Cohuet S et al. 2006 [6] | 2003-2004 | 3 | 458 | *Pf*DHFR, *Pf*DHPS | Moderate |
| 6 | Deutsch-Feldman M et al. 2019 [7] | 2013-2014, 2007 | Unkown | 852 | *Pf*DHFR, *Pf*CRT | Moderate |
| 7 | Kamau E et al. 2015 [8] | 2013-2014 | 1 | 82 | *Pf*K13 | Moderate |
| 8 | Kayiba NK et al. 2021 [9] | 2018-2019 | 1 | 844 | *Pf*DHPS, *Pf*DHFR | Moderate |
| 9 | Leroy D et al. 2019 [10] | 2014-2015 | 1 | 5 | *Pf*K13 | Moderate |
| 10 | Menard D et al. 2016 [11] | 2009-2014 | Unkown | 1288 | *Pf*K13 | Moderate |
| 11 | Mens PF et al. 2008 [12] | 2003-2004 | 1 | 78 | *Pf*DHPS | Moderate |
| 12 | Miotto O et al. 2015 [13] | 2009-2010 | 1 | 115 | *Pf*K13 | Moderate |
| 13 | Mobula L et al. 2009 [14] | 2008 | 1 | 142 | *Pf*CRT, *Pf*MDR1, *Pf*DHFR, *Pf*DHPS | Moderate |
| 14 | Moriarty LF et al. 2021 [15] | 2017-2018 | 5 | 523 | *Pf*CRT, *Pf*K13, *Pf*MDR1 | Moderate |
| 15 | Mvumbi DM et al. 2013[16] | 2010 | 1 | 145 | *Pf*CRT | Moderate |
| 16 | Mvumbi DM et al. 2017 [17] | 2014 | 6 | 280 | *Pf*K13 | Moderate |
| 17 | Nkoli Mandoko P et al. 2018 [18] | 2014-2015 | 2 | 2030 | *Pf*DHFR, *Pf*DHPS | Moderate |
| 18 | Nundu SS et al. 2022 [19] | 2019 | 1 | 229 | *Pf*K13, *Pf*MDR1, *Pf*DHFR, *Pf*DHPS, *Pf*CRT | Moderate |
| 19 | Ruh E et al. 2018 [20] | 2014 | 1 | 48 | *Pf*DHFR, *Pf*DHPS | Moderate |
| 20 | Severini C et al. 2006 [21] | 2000 | 1 | 27 | *Pf*CRT | Moderate |
| 21 | Swarthout TD et al. 2006 [22] | 2003 | 1 | 217 | *Pf*DHFR, *Pf*DHPS | Moderate |
| 22 | Taylor SM et al. 2014 [23] | 2007 | 11 | 179 | *Pf*DHPS | Moderate |
| 23 | Taylor SM et al. 2015 [24] | 2007 | 3 | 151 | *Pf*K13 | Moderate |
| 24 | van Lenthe M et al. 2019 [25] | 2017 | 4 | 1086 | *Pf*DHFR, *Pf*DHPS | Moderate |
| 25 | Wilson PE et al. 2005 [26] | 2002 | 1 | 56 | *Pf*CRT | Moderate |
| 26 | Yobi DM et al. 2020a [27] | 2017 | 10 | 1070 | *Pf*K13 | Moderate |
| 27 | Yobi DM et al. 2020b [28] | 2017 | 10 | 1070 | *Pf*CRT | Moderate |
| 28 | Yobi DM et al. 2021 [29] | 2018-2019 | 6 | 474 | *Pf*CRT, *Pf*K13, *Pf*MDR1 | Moderate |
| 29 | Yobi DM et al. 2022 [30] | 2019-2020 | 10 | 1087 | *Pf*CRT, *Pf*K13 | Moderate |

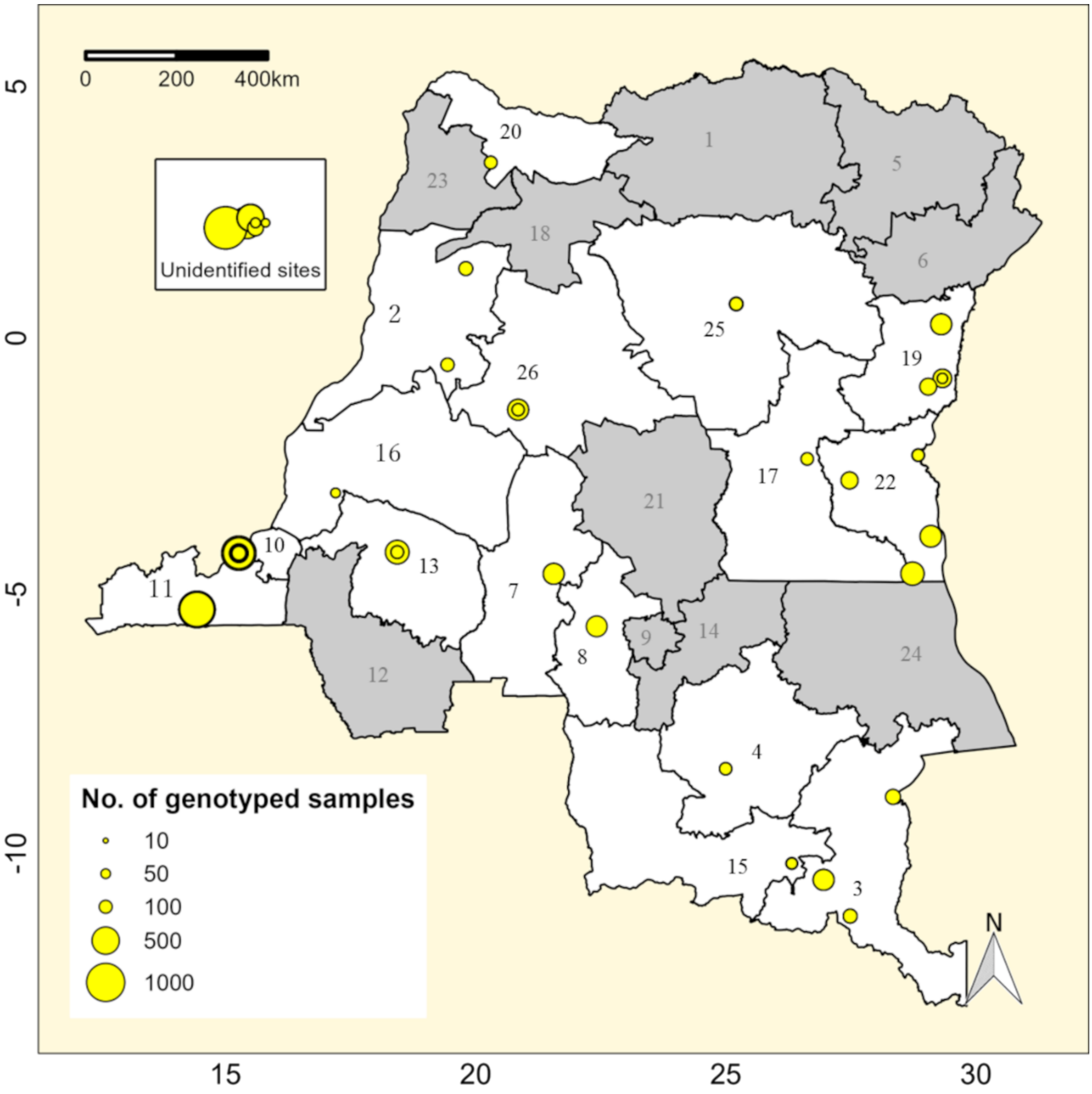


**Figure S1. Geographic locations of collection sites in primary studies.**

The location of each primary survey has been uploaded onto this map. Shaded areas represent the country's provinces that were not surveyed during this review. Yellow circles reflect individual surveys conducted at different locations with a diameter proportional to the size of the samples that were successfully investigated for molecular markers of antimalarial drug resistance. The numbers written on the map represent the 26 provinces of the country as follows: Bas-Uele : 1; Equateur : 2; Haut-Katanga : 3; Haut-Lomami : 4; Haut-Uele : 5; Ituri : 6; Kasai : 7; Kasai-Central : 8; Kasai-Oriental : 9; Kinshasa :10; Kongo-Central : 11; Kwango : 12; Kwilu : 13; Lomami : 14; Lualaba : 15; Mai-Ndombe : 16; Maniema : 17; Mongala : 18; Nord-Kivu : 19; Nord-Ubangi : 20; Sankuru : 21; Sud-Kivu : 22; Sud-Ubangi : 23; Tanganyika : 24; Tshopo : 25; and Tshuapa : 26.

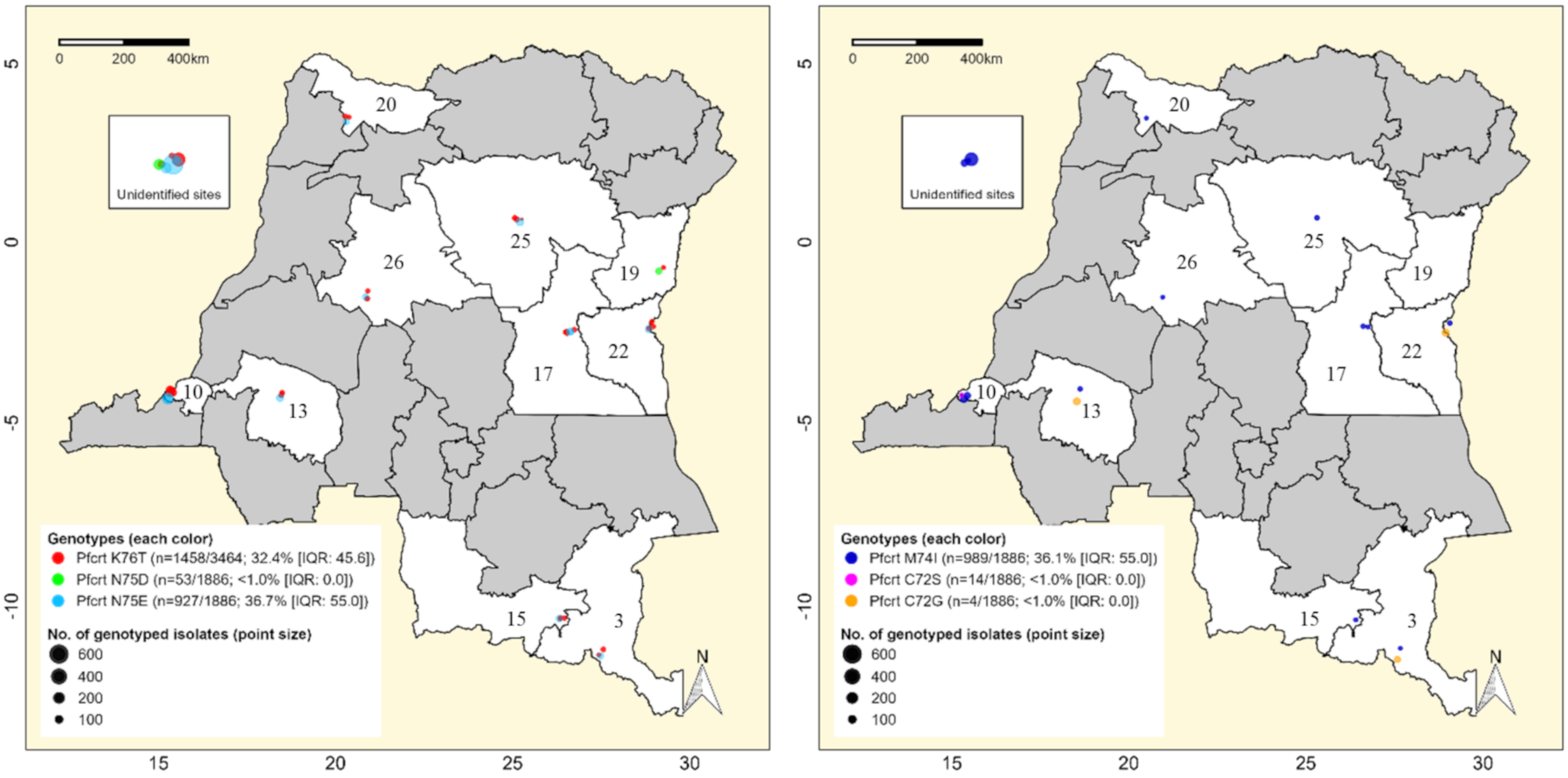


**Figure S2. Distribution of parasites carrying different *Pf*CRT mutations.**

Circles displayed on these maps reflect surveys that reported at least one parasite carrying a mutation on *Pf*CRT 72-76 codon-positions. The diameter of each circle is proportional to the number of isolates for which the corresponding *Pf*CRT 72-76 codon-position has been successfully genotyped. Individual mutations are reported with absolute frequencies and relative proportions across different surveyed sites. Shaded areas reflect provinces that have not been monitored for parasites harboring *Pf*CRT 72-76 mutations. The numbers written on the map represent the 26 provinces of the country as follows: Haut-Katanga : 3; Kinshasa :10; Kwilu : 13; Lualaba : 15; Maniema : 17; Nord-Kivu : 19; Nord-Ubangi : 20; Sud-Kivu : 22; Tshopo : 25; and Tshuapa : 26.

**Table S5. Evolution of the frequency of parasite carrying a *Pf*CRT K76T mutation in the DRC.***

| Sampling year | n | m | Median % of *Pf*CRT K76T isolates (IQR) |
|---|---|---|---|
| 2000 | 27 | 27 | 100 (0.0) |
| 2002 | 56 | 52 | 92.9 (0.0) |
| 2007 | 841 | 461 | 55.0 (0.4) |
| 2008 | 105 | 88 | 83.8 (0.0) |
| 2010 | 198 | 145 | 73.2 (0.0) |
| 2017 | 849 | 218 | 11.3 (40.5) |
| 2018 | 335 | 139 | 33.6 (22.1) |
| 2019 | 1053 | 199 | 13.3 (23.2) |

(*) n: No. of genotyped isolates; m: No. of detected mutants

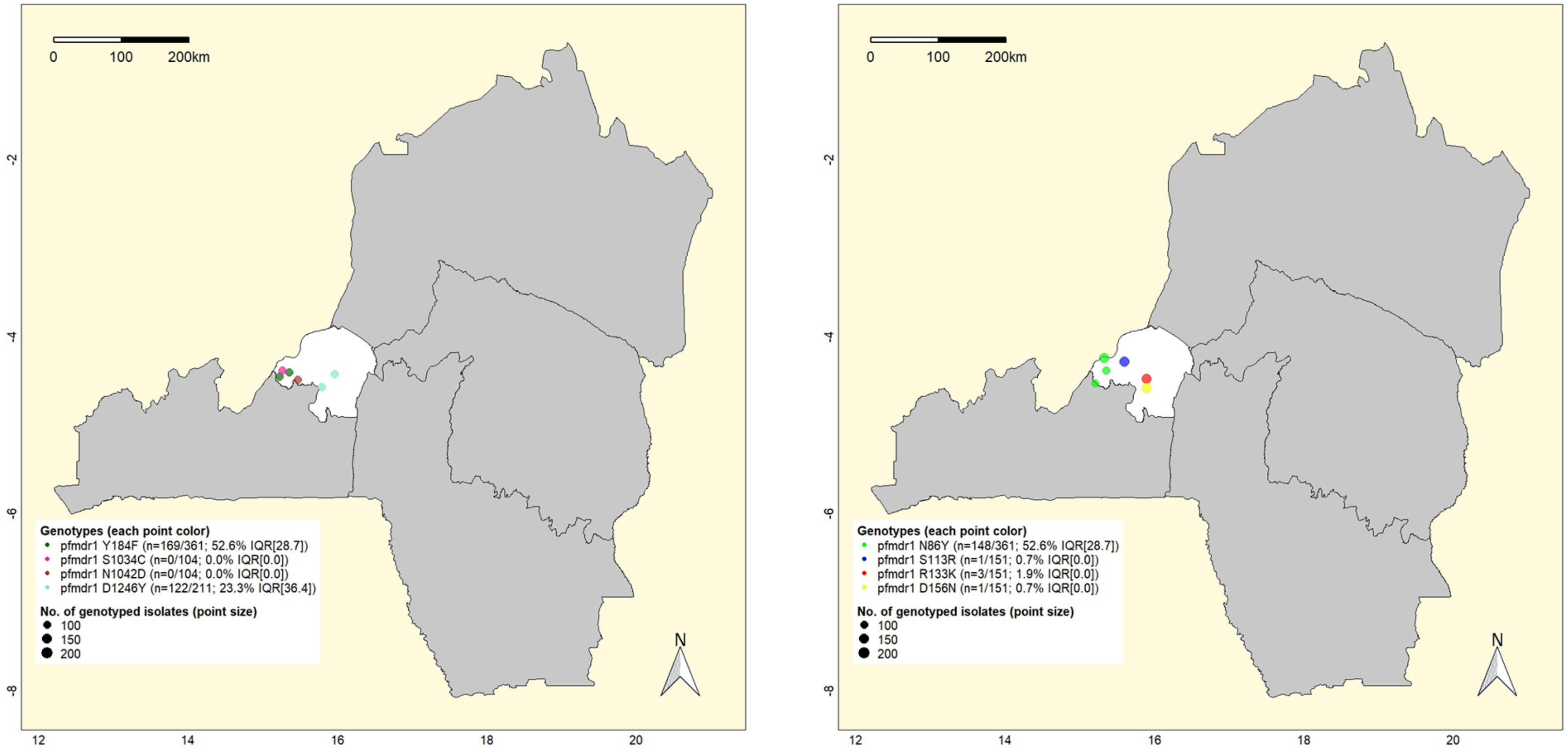


**Figure S3. Distribution of parasites carrying *Pfmdr*1 SNPs in the DRC.**

Each survey having explored parasites carrying different *Pfmdr1* mutations were uploaded on these two maps. These parasites were explored only in Kinshasa, the country&s capital city (shown here as an unshaded area). Circles represent surveys conducted at different locations with a diameter proportional to the sample size of isolates that have been successfully analyzed for the corresponding alteration. The colors differentiate the different *Pfmdr1* mutations sought and for which the median frequencies and the respective interquartile ranges are presented.

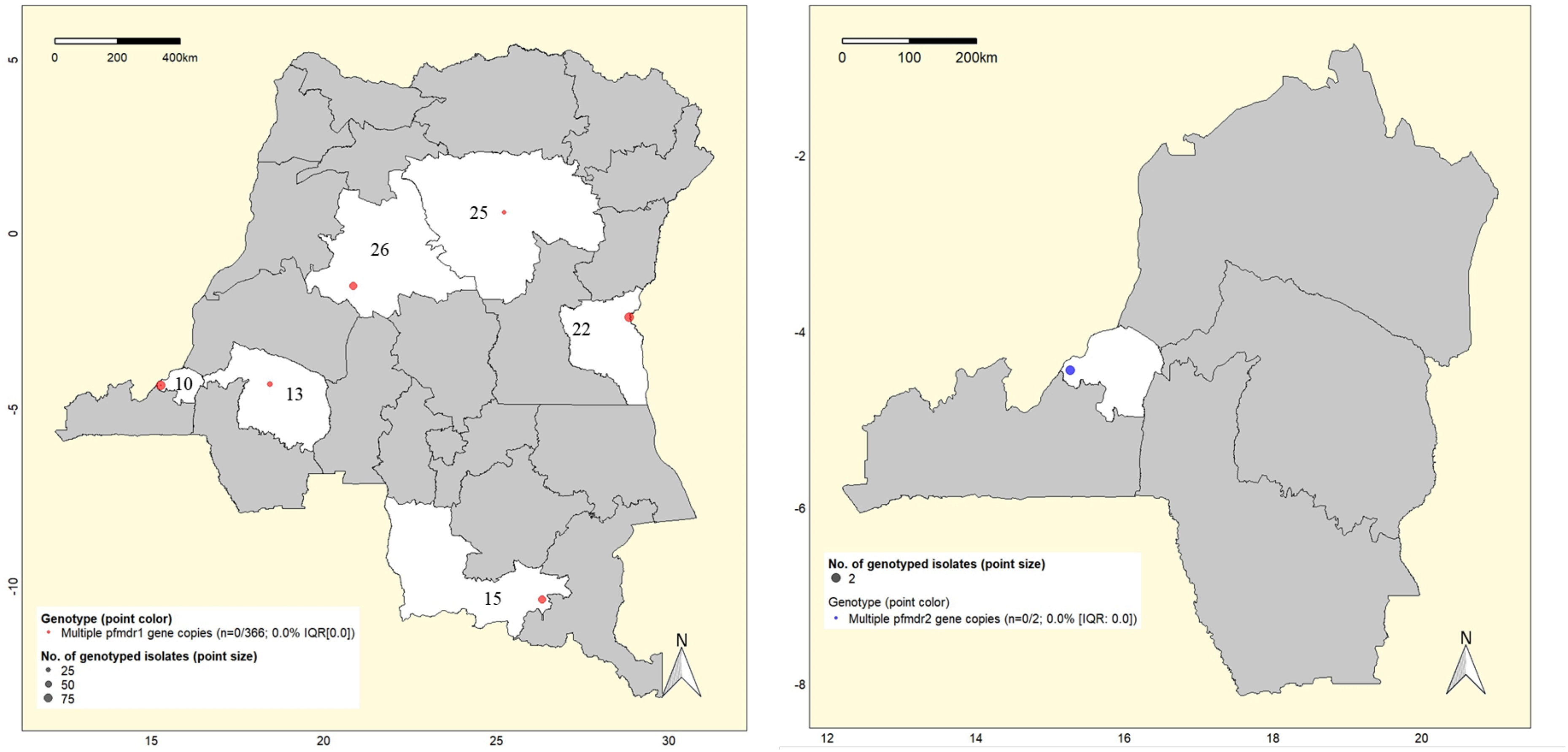


**Figure S4. Distribution of parasites genotyped for *Pfmdr*1 and *Pfmdr2* gene copy number alterations in the DRC.**

Each survey having genotyped parasites for potential alterations in *Pfmdr1* and *Pfmdr1* gene copy numbers were uploaded on these two maps. Unshaded areas are provinces that have been explored for these parasites (Kinshasa :10; Kwilu : 13; Lualaba : 15; Sud-Kivu : 22; Tshopo : 25; and Tshuapa : 26). Circles reflect surveys conducted at different locations with a diameter proportional to the sample size of isolates that have been successfully analyzed for the gene alterations. The colors differentiate the different genes explored for which the number of isolates, the median frequencies and the respective interquartile ranges are presented.

**Table S6. Overall proportions of parasites carrying non-synonymous mutations of the *Pf*K13 in the DRC***

| Locus | NS mutation | n | m | % |
|---|---|---|---|---|
| 92 | K92N | 79 | 2 | 2,5 |
| 149 | T149S | 79 | 4 | 5,1 |
| 189 | K189T | 79 | 20 | 25,3 |
| 255 | R255K | 79 | 4 | 5,1 |
| 472 | M472I | 5383 | 1 | 0,0 |
| 472 | M472L | 5383 | 1 | 0,0 |
| 473 | C473Y | 5383 | 1 | 0,0 |
| 476 | M476K | 5383 | 1 | 0,0 |
| 477 | S477Y | 5383 | 1 | 0,0 |
| 495 | F495L | 5383 | 2 | 0,0 |
| 498 | N498I | 5383 | 1 | 0,0 |
| 506 | F506L | 5383 | 1 | 0,0 |
| 507 | E507V | 5383 | 1 | 0,0 |
| 509 | E509D | 5383 | 2 | 0,0 |
| 516 | D516A | 5383 | 1 | 0,0 |
| 516 | D516E | 5383 | 1 | 0,0 |
| 520 | V520A | 5383 | 4 | 0,1 |
| 522 | S522C | 5383 | 2 | 0,0 |
| 523 | N523T | 5383 | 1 | 0,0 |
| 532 | C532S | 5383 | 1 | 0,0 |
| 534 | V534A | 5383 | 4 | 0,1 |
| 538 | G538S | 5383 | 1 | 0,0 |
| 554 | N554K | 5383 | 2 | 0,0 |
| 557 | A557S | 5383 | 3 | 0,1 |
| 561 | R561H | 5383 | 2 | 0,0 |
| 567 | E567K | 5383 | 1 | 0,0 |
| 568 | V568M | 5383 | 1 | 0,0 |
| 569 | A569T | 5383 | 1 | 0,0 |
| 576 | S576T | 5383 | 1 | 0,0 |
| 578 | A578S | 5383 | 10 | 0,2 |
| 584 | D584E | 5383 | 2 | 0,0 |
| 588 | Y588C | 5383 | 1 | 0,0 |
| 596 | E596K | 5383 | 1 | 0,0 |
| 613 | Q613E | 5383 | 3 | 0,1 |

(*) n: No. of genotyped isolates; m: No. of detected mutants; %: overall proportion of mutants; loci beyond 442 are located in the propeller domain.

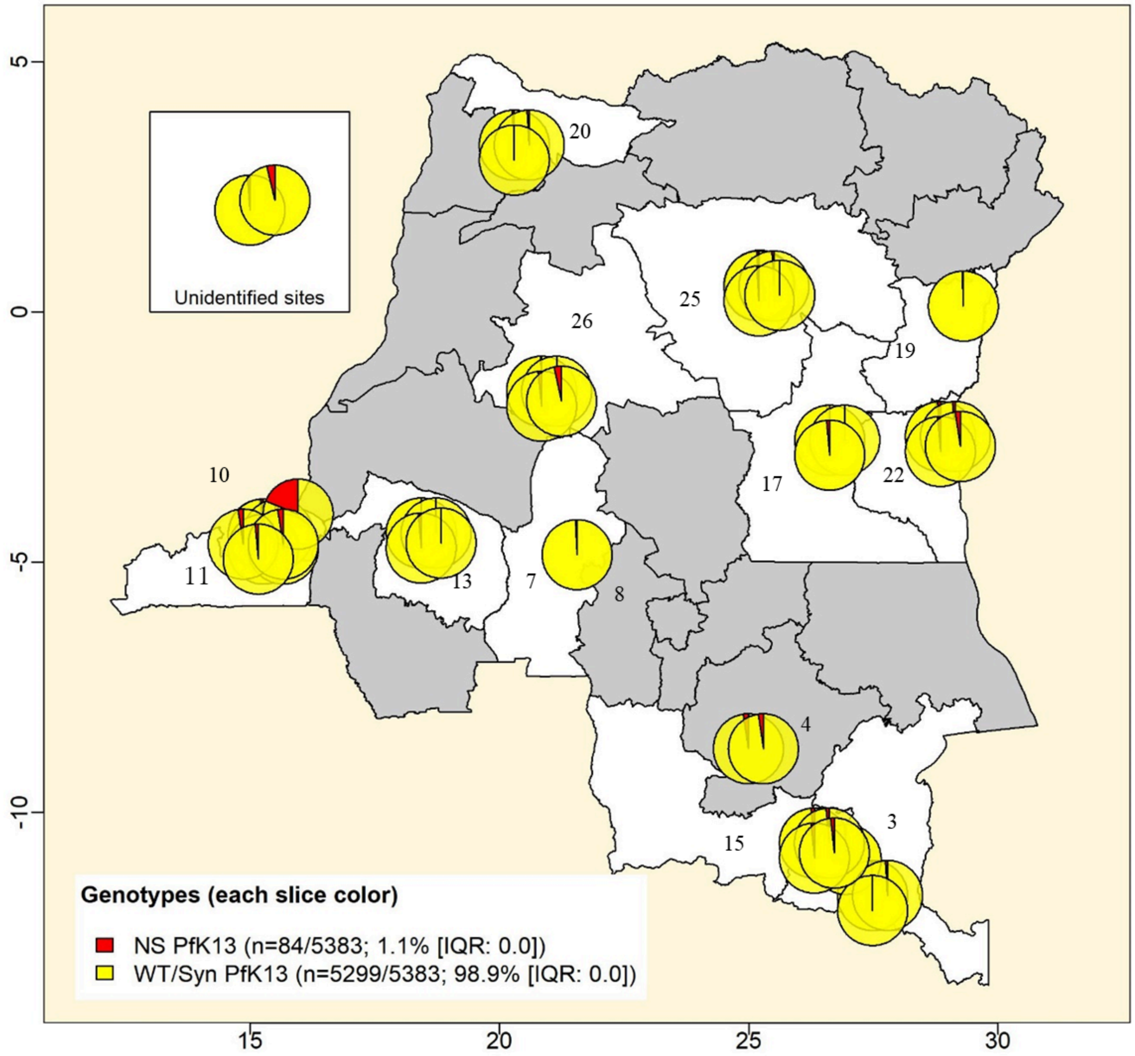


**Figure S5 Distribution of *Plasmodium* parasites carrying non-synonymous *Pf*K13 mutations in the DRC.**

The pie charts displayed on these maps represent the proportions of isolates carrying at least one non-synonymous *Pf*K13 mutation (NS, portion colored in red) and those of isolates carrying a wild-type *Pf*K13 sequence or with only synonyms mutations (WT/Syn, portion colored in yellow). The numbers written on the map represent differents provinces that have been explored so far for these parasites: Haut-Katanga : 3; Haut-Lomami : 4; Kasai : 7; Kasai-Central : 8; Kinshasa :10; Kongo-Central : 11; Maniema : 17; Nord-Kivu : 19; Nord-Ubangi : 20; Sud-Kivu : 22; Tshopo : 25; and Tshuapa : 26.

**Table S7. Overall proportions of parasites encoding different *Pfdhfr-Pfdhps* haplotypes in the DRC***

| Haplotype | n | m | % |
|---|---|---|---|
| **Wild-type haplotype** | | | |
| NCS-AK (i.e., *Pfdhfr* N51, C59, S108; *Pfdhps* A437, K540) | 2092 | 9 | 0,7 |
| **Mutant haplotypes** | | | |
| IRN-AE (i.e., *Pfdhfr* N51I, C59R, S108N; *Pfdhps* A437, K540E) | 2092 | 1 | 0,0 |
| NCS-GK (i.e., *Pfdhfr* N51, C59, S108; *Pfdhps* A437G, K540) | 2092 | 1 | 0,0 |
| NCN-GK (i.e., *Pfdhfr* N51, C59, S108N; *Pfdhps* A437G, K540) | 2092 | 2 | 0,1 |
| NRN-AK (i.e., *Pfdhfr* N51, C59R, S108N; *Pfdhps* A437, K540) | 2092 | 2 | 0,1 |
| NCS-GE (i.e., *Pfdhfr* N51, C59, S108; *Pfdhps* A437G, K540E) | 2092 | 3 | 0,1 |
| ICN-AK (i.e., *Pfdhfr* N51I, C59, S108N; *Pfdhps* A437, K540) | 2092 | 6 | 0,3 |
| ICN-GE (i.e., *Pfdhfr* N51I, C59, S108N; *Pfdhps* A437G, K540E) | 2092 | 16 | 0,8 |
| NRN-GK (i.e., *Pfdhfr* N51, C59R, S108N; *Pfdhps* A437G, K540) | 2092 | 17 | 0,8 |
| IRN-AK (i.e., *Pfdhfr* N51I, C59R, S108N; *Pfdhps* A437, K540) | 2092 | 19 | 0,9 |
| ICN-GK (i.e., *Pfdhfr* N51I, C59, S108N; *Pfdhps* A437G, K540) | 2092 | 139 | 6,6 |
| IRN-GE (i.e., *Pfdhfr* N51I, C59R, S108N; *Pfdhps* A437G, K540E) | 2092 | 320 | 13,1 |
| IRN-GK (i.e., *Pfdhfr* N51I, C59R, S108N; *Pfdhps* A437G, K540) | 2092 | 1018 | 59,3 |
| Unspecified | 2092 | 539 | 21,8 |

(*) n: No. of genotyped isolates; m: No. of detected mutants; %: median proportion of mutants

## References


1. Takkouche B, Norman G: **PRISMA statement**. *Epidemiology* 2011, **22**(1):128.
2. Alker AP, Kazadi WM, Kutelemeni AK, Bloland PB, Tshefu AK, Meshnick SR: **dhfr and dhps genotype and sulfadoxine‐pyrimethamine treatment failure in children with falciparum malaria in the Democratic Republic of Congo**. *Tropical Medicine & International Health* 2008, **13**(11):1384-1391.
3. Antonia AL, Taylor SM, Janko M, Emch M, Tshefu AK, Meshnick SR: **A cross-sectional survey of Plasmodium falciparum pfcrt mutant haplotypes in the Democratic Republic of Congo**. *The American journal of tropical medicine and hygiene* 2014, **90**(6):1094-1097.
4. Baraka V, Delgado-Ratto C, Nag S, Ishengoma DS, Madebe RA, Mavoko HM, Nabasumba C, Lutumba P, Alifrangis M, Van Geertruyden J-P: **Different origin and dispersal of sulfadoxine-resistant Plasmodium falciparum haplotypes between Eastern Africa and Democratic Republic of Congo**. *International journal of antimicrobial agents* 2017, **49**(4):456-464.
5. Baraka V, Mavoko HM, Nabasumba C, Francis F, Lutumba P, Alifrangis M, Van Geertruyden J-P: **Impact of treatment and re-treatment with artemether-lumefantrine and artesunate-amodiaquine on selection of Plasmodium falciparum multidrug resistance gene-1 polymorphisms in the Democratic Republic of Congo and Uganda**. *PLoS One* 2018, **13**(2):e0191922.
6. Cohuet S, Bonnet M, Van Herp M, Van Overmeir C, D'Alessandro U, Guthmann J-P: **molecular markers associated with Plasmodium falciparum resistance to sulfadoxine-pyrimethamine in the Democratic Republic of Congo**. 2006.
7. Deutsch-Feldman M, Aydemir O, Carrel M, Brazeau NF, Bhatt S, Bailey JA, Kashamuka M, Tshefu AK, Taylor SM, Juliano JJ: **The changing landscape of Plasmodium falciparum drug resistance in the Democratic Republic of Congo**. *BMC infectious diseases* 2019, **19**(1):1-10.
8. Kamau E, Campino S, Amenga-Etego L, Drury E, Ishengoma D, Johnson K, Mumba D, Kekre M, Yavo W, Mead D: **K13-propeller polymorphisms in Plasmodium falciparum parasites from sub-Saharan Africa**. *The Journal of infectious diseases* 2015, **211**(8):1352-1355.
9. Kayiba NK, Yobi DM, Kouoneyou VRT, Mvumbi DM, Kabututu PZ, Devleesschauwer B, Sompwe EM, DeMol P, Hayette M-P, Mvumbi GL: **Evaluation of the usefulness of intermittent preventive treatment of malaria in pregnancy with sulfadoxine-pyrimethamine in a context with increased resistance of Plasmodium falciparum in Kingasani Hospital, Kinshasa in the Democratic Republic of Congo**. *Infection, Genetics and Evolution* 2021:105009.
10. Leroy D, Macintyre F, Adoke Y, Ouoba S, Barry A, Mombo-Ngoma G, Ngomo JMN, Varo R, Dossou Y, Tshefu AK: **African isolates show a high proportion of multiple copies of the Plasmodium falciparum plasmepsin-2 gene, a piperaquine resistance marker**. *Malaria journal* 2019, **18**(1):1-11.
11. Ménard D, Khim N, Beghain J, Adegnika AA, Shafiul-Alam M, Amodu O, Rahim-Awab G, Barnadas C, Berry A, Boum Y: **A worldwide map of Plasmodium falciparum K13-propeller polymorphisms**. *New England Journal of Medicine* 2016, **374**(25):2453-2464.
12. Mens PF, van Overmeir C, Bonnet M, Dujardin JC, d'Alessandro U: **Real-time PCR/MCA assay using fluorescence resonance energy transfer for the genotyping of resistance related DHPS-540 mutations in Plasmodium falciparum**. *Malar J* 2008, **7**:48.
13. Miotto O, Amato R, Ashley EA, MacInnis B, Almagro-Garcia J, Amaratunga C, Lim P, Mead D, Oyola SO, Dhorda M: **Genetic architecture of artemisinin-resistant Plasmodium falciparum**. *Nature genetics* 2015, **47**(3):226-234.
14. Mobula L, Lilley B, Tshefu AK, Rosenthal PJ: **Resistance-mediating polymorphisms in Plasmodium falciparum infections in Kinshasa, Democratic Republic of the Congo**. *The American journal of tropical medicine and hygiene* 2009, **80**(4):555-558.
15. Moriarty LF, Nkoli PM, Likwela JL, Mulopo PM, Sompwe EM, Rika JM, Mavoko HM, Svigel SS, Jones S, Ntamabyaliro NY *et al*: **Therapeutic Efficacy of Artemisinin-Based Combination Therapies in Democratic Republic of the Congo and Investigation of Molecular Markers of Antimalarial Resistance**. *The American journal of tropical medicine and hygiene* 2021, **105**(4):1067-1075.
16. Mvumbi DM, Boreux R, Sacheli R, Lelo M, Lengu B, Nani-Tuma S, Melin P, Ntumba K, Lunganza K, DeMol P *et al*: **Assessment of pfcrt 72-76 haplotypes eight years after chloroquine withdrawal in Kinshasa,**

**Democratic Republic of Congo**. *Malar J* 2013, **12**:459.
17. Mvumbi DM, Bobanga TL, Kayembe JN, Mvumbi GL, Situakibanza HN, Benoit-Vical F, Melin P, De Mol P, Hayette MP: **Molecular surveillance of Plasmodium falciparum resistance to artemisinin-based combination therapies in the Democratic Republic of Congo**. *PLoS One* 2017, **12**(6):e0179142.
18. Nkoli Mandoko P, Rouvier F, Matendo Kakina L, Moke Mbongi D, Latour C, Losimba Likwela J, Ngoyi Mumba D, Bi Shamamba SK, Tamfum Muyembe J-J, Muepu Tshilolo L: **Prevalence of Plasmodium falciparum parasites resistant to sulfadoxine/pyrimethamine in the Democratic Republic of the Congo: emergence of highly resistant pfdhfr/pfdhps alleles**. *Journal of Antimicrobial Chemotherapy* 2018, **73**(10):2704-2715.
19. Nundu SS, Culleton R, Simpson SV, Arima H, Chitama BA, Muyembe JJ, Ahuka S, Kaneko O, Mita T, Yamamoto T: **Identification of polymorphisms in genes associated with drug resistance in Plasmodium falciparum isolates from school-age children in Kinshasa, Democratic Republic of Congo**. *Parasitology international* 2022, **88**:102541.
20. Ruh E, Bateko JP, Imir T, Taylan-Ozkan A: **Molecular identification of sulfadoxine-pyrimethamine resistance in malaria infected women who received intermittent preventive treatment in the Democratic Republic of Congo**. *Malar J* 2018, **17**(1):17.
21. Severini C, Menegon M, Sannella AR, Paglia MG, Narciso P, Matteelli A, Gulletta M, Caramello P, Canta F, Xayavong MV: **Prevalence of pfcrt point mutations and level of chloroquine resistance in Plasmodium falciparum isolates from Africa**. *Infection, Genetics and Evolution* 2006, **6**(4):262-268.
22. Swarthout TD, van den Broek IV, Kayembe G, Montgomery J, Pota H, Roper C: **Artesunate + amodiaquine and artesunate + sulphadoxine-pyrimethamine for treatment of uncomplicated malaria in Democratic Republic of Congo: a clinical trial with determination of sulphadoxine and pyrimethamine-resistant haplotypes**. *Tropical medicine & international health : TM & IH* 2006, **11**(10):1503-1511.
23. Taylor SM, Antonia AL, Harrington WE, Goheen MM, Mwapasa V, Chaluluka E, Fried M, Kabyemela E, Madanitsa M, Khairallah C *et al*: **Independent lineages of highly sulfadoxine-resistant Plasmodium falciparum haplotypes, eastern Africa**. *Emerging infectious diseases* 2014, **20**(7):1140-1148.
24. Taylor SM, Parobek CM, DeConti DK, Kayentao K, Coulibaly SO, Greenwood BM, Tagbor H, Williams J, Bojang K, Njie F *et al*: **Absence of putative artemisinin resistance mutations among Plasmodium falciparum in Sub-Saharan Africa: a molecular epidemiologic study**. *J Infect Dis* 2015, **211**(5):680-688.
25. van Lenthe M, van der Meulen R, Lassovski M, Ouabo A, Bakula E, Badio C, Cibenda D, Okell L, Piriou E, Grignard L *et al*: **Markers of sulfadoxine-pyrimethamine resistance in Eastern Democratic Republic of Congo; implications for malaria chemoprevention**. *Malar J* 2019, **18**(1):430.
26. Wilson PE, Kazadi W, Kamwendo DD, Mwapasa V, Purfield A, Meshnick SR: **Prevalence of pfcrt mutations in Congolese and Malawian Plasmodium falciparum isolates as determined by a new Taqman assay**. *Acta tropica* 2005, **93**(1):97-106.
27. Yobi DM, Kayiba NK, Mvumbi DM, Boreux R, Bontems S, Kabututu PZ, De Mol P, Speybroeck N, Mvumbi GL, Hayette MP: **The lack of K13-propeller mutations associated with artemisinin resistance in Plasmodium falciparum in Democratic Republic of Congo (DRC)**. *PLoS One* 2020, **15**(8):e0237791.
28. Yobi DM, Kayiba NK, Mvumbi DM, Boreux R, Kabututu PZ, Situakibanza HNT, Likwela JL, De Mol P, Okitolonda EW, Speybroeck N *et al*: **Molecular surveillance of anti-malarial drug resistance in Democratic Republic of Congo: high variability of chloroquinoresistance and lack of amodiaquinoresistance**. *Malar J* 2020, **19**(1):121.
29. Yobi DM, Kayiba NK, Mvumbi DM, Boreux R, Kabututu PZ, Situakibanza HNT, Umesumbu SE, De Mol P, Speybroeck N, Mvumbi GL *et al*: **Assessment of Plasmodium falciparum anti-malarial drug resistance markers in pfk13-propeller, pfcrt and pfmdr1 genes in isolates from treatment failure patients in Democratic Republic of Congo, 2018-2019**. *Malar J* 2021, **20**(1):144.
30. Yobi DM, Kayiba NK, Mvumbi DM, Boreux R, Kabututu PZ, Akilimali PZ, Situakibanza HNT, De Mol P, Speybroeck N, Mvumbi GL *et al*: **Biennial surveillance of Plasmodium falciparum anti-malarial drug resistance markers in Democratic Republic of Congo, 2017 and 2019**. *BMC Infect Dis* 2022, **22**(1):145.
